# Intrauterine exposure to maternal diabetes and the risk of developing epilepsy in children: a national cohort study of 2.3 million children

**DOI:** 10.1186/s12916-026-04696-0

**Published:** 2026-02-14

**Authors:** Muhammad Zakir Hossin, Martina Persson, Marte-Helene Bjørk, Olof Stephansson, Neda Razaz

**Affiliations:** 1 Department of Medicine, Solna, Division of Clinical Epidemiology, Karolinska Institutet, Stockholm, SE-171 76 Sweden; 2 School of Population and Public Health, Human Early Learning Partnership, The University of British Columbia, Vancouver, Canada; 3 Department of Clinical Science and Education, Karolinska Institutet Södersjukhuset, Stockholm, Sweden; 4 Department of Clinical Medicine, University of Bergen, Bergen, Norway; 5 Department of Neurology, Haukeland University Hospital, Bergen, Norway; 6 Department of Women’s Health, Division of Obstetrics, Karolinska University Hospital, Stockholm, Sweden

**Keywords:** Type 1 diabetes, Type 2 diabetes, Gestational diabetes, Epilepsy, Negative-control, Sequential mediation

## Abstract

**Background:**

Approximately half of the epilepsy cases lack a documented cause. Despite growing evidence linking maternal diabetes to pregnancy complications and impaired child neurodevelopment, little is known about its potential association with child epilepsy. This study aims to determine whether intrauterine exposure to maternal pre-existing type 1 diabetes mellitus (T1DM), type 2 diabetes mellitus (T2DM), or gestational diabetes mellitus (GDM) increases the risk of epilepsy in children.

**Methods:**

This study included live-born children without major malformations in Sweden from 1998 to 2021. Children were followed from birth until epilepsy diagnosis, death, emigration, or October 2023. Child epilepsy, maternal T1DM, T2DM, GDM, and a comprehensive set of covariates were identified through linked national health registers. Multivariable Cox proportional hazards regression was used to examine the association between maternal diabetes and child epilepsy, estimating adjusted hazard ratios (aHRs) and 95% confidence intervals (CIs). Analyses also included paternal T1DM and T2DM as negative control exposures to assess potential genetic confounding. Preeclampsia, preterm birth, and birth asphyxia were evaluated as mediators, using a sequential mediation approach.

**Results:**

Of the 2,305,051 children, 14,283 (0.6%) were exposed to maternal T1DM, 3,833 (0.2%) to T2DM, and 36,388 (1.6%) to GDM. Over a median follow-up of 13 years (range 0 to 25.8 years), 18,968 were diagnosed with epilepsy, corresponding to an incidence rate of 63.7 per 100,000 person-years. Compared with no maternal diabetes, maternal T1DM and T2DM were associated with increased hazards of epilepsy in children, with aHRs of 1.30 (95% CI 1.11–1.52) and 1.41 (95% CI 1.01–1.97), respectively. Preterm birth and birth asphyxia jointly mediated nearly half of the total effect of T1DM (HR^Indirect Effect^:1.14, 95% CI: 1.11, 1.17) and 30% of the total effect of T2DM (HR^Indirect Effect^:1.11, 95% CI: 1.04, 1.18). Paternal T1DM and T2DM, as well as maternal GDM, were not associated with epilepsy risk.

**Conclusions:**

In this large cohort study, maternal T1DM and T2DM were associated with an increased risk of epilepsy in children. These associations are likely influenced by intrauterine mechanisms and were partially mediated by preterm birth and asphyxia-related conditions at birth.

**Supplementary Information:**

The online version contains supplementary material available at 10.1186/s12916-026-04696-0.

## Summary box

What is already known on the topic

- Nearly half of the epilepsy cases lack a documented cause, and pregnancy-related factors may contribute to risk.
- Maternal pre-gestational diabetes and gestational diabetes mellitus (GDM) have been linked to neurodevelopmental disorders in children.
- Little is known about the relationship between in utero exposure to maternal diabetes and offspring epilepsy risk.

What this study adds

- Using nationwide register data on over 2.3 million children born in Sweden between 1998 and 2021, this study provides the most comprehensive evidence to date on maternal diabetes and child epilepsy.
- Maternal pre-gestational type 1 (T1DM) and type 2 diabetes (T2DM) were associated with increased risk of epilepsy in children, while GDM was not.
- A family-based design using paternal type 1 and type 2 diabetes as negative controls supported an intrauterine origin for these associations. Sequential mediation analyses involving key obstetric and neonatal complications revealed that preterm birth and birth-asphyxia each mediated a substantial proportion of the observed risks.

How this study might affect research, practice, or policy

- This study adds valuable evidence to early-life determinants of epilepsy, identifying maternal pre-existing T1DM and T2DM as potential risk factors.
- Prevention and intervention strategies targeting preterm births and asphyxia-related neonatal conditions may help reduce epilepsy risk associated with maternal diabetes. However, addressing maternal diabetes directly may be a more effective intervention, given its potential direct effects through unexplored pathways.
- The lack of an association between GDM and child epilepsy implies that timely management of maternal hyperglycaemia may benefit long-term child neurodevelopment, though further research is needed.

## Background

Epilepsy is a long-term neurological disease characterized by unprovoked seizures and affects nearly 50 million people around the world [1, 2]. The etiologic causes and mechanisms leading to epilepsy are not clearly understood [3]. Previous research has linked prenatal risk factors, such as maternal smoking, preeclampsia, infection, as well as preterm birth and low birth weight, to an increased risk of epilepsy [4]. However, the role of pre-existing maternal diseases, including diabetes, in the development of epilepsy is not well-examined.

Diabetes is a common chronic condition among people of childbearing age, and is associated with a broad spectrum of maternal and fetal complications [5, 6]. Pregnancies complicated by diabetes exhibit higher rates of preeclampsia, large for gestational age (LGA), preterm birth, and asphyxia-related neonatal complications compared with the general population [7–10]. Additionally, maternal type 1 diabetes mellitus (T1DM) and type 2 diabetes mellitus (T2DM) have been linked to child cognitive impairment and neurodevelopmental disorders [11–13], including autism spectrum disorder (ASD) [14, 15], attention-deficit/ hyperactivity disorder (ADHD) [16], intellectual disability [17], cerebral palsy [18], and neonatal seizures [19]. An observational study in Taiwan identified a higher incidence of epilepsy and other neurodevelopmental disorders among children born to mothers with pre-gestational diabetes. However, that study did not adjust for key confounders, had limited statistical power, and did not explicitly examine potential mediating pathways [20], underscoring the need for further research focused specifically on epilepsy.

Maternal hyperglycaemia during pregnancy may elevate the risk of epilepsy in children by impacting the fetal central nervous system [12, 21, 22]. However, epilepsy can typically have a focal or generalized origin and is likely caused by genetic factors, environmental influences, or their interactions. Focal epilepsy more often results from acquired causes, such as birth asphyxia, while generalized epilepsy in general is assumed to have genetic causes, though a monogenetic cause is rare [4, 23, 24]. Evidence from polygenic risk score analysis and familial aggregation research indicates that the heritability of epilepsy indeed differs by its subtypes, with generalized epilepsy showing a stronger genetic predisposition than focal epilepsy [24, 25]. If maternal diabetes and child epilepsy share common genetic elements [26, 27], any observed association between them could be prone to genetic confounding.

This large Swedish cohort study primarily aimed to determine whether intrauterine exposure to maternal pre-existing T1DM, T2DM, or GDM is associated with an increased risk of epilepsy in children. Additionally, the study sought to evaluate whether any observed association could be attributed to unmeasured genetic or familial confounding, by employing paternal diabetes as a negative control, and to investigate mediation through key obstetric and neonatal complications, namely preeclampsia, preterm birth, birth asphyxia, and birthweight for gestational age.

## Methods

### Data sources

Data for this cohort study were primarily obtained from the Swedish Medical Birth Register, which has recorded more than 98% of all births in Sweden since 1973 and is widely regarded as a high-quality resource for perinatal research [28]. Linkage between the Medical Birth Register and other national registers, including the National Patient Register (inpatient and outpatient specialist care) [29, 30], the Cause of Death Register [31], the Total Population Register [32], the Education Register [33], the Prescription Drug Register (since July 2005) [34], and the Multi-generation Register [35] (Additional File 1: Supplementary Methods Text S1), was achieved using the person-unique identity number assigned at birth (or upon immigration for foreign-born parents). The study received approval from the Regional Ethics Committee in Stockholm, Sweden.

### Study population

In the Medical Birth Register, we identified 2,481,137 singletons, live-born at ≥ 22 completed gestational weeks in Sweden between January 1, 1998 and December 31, 2021. Exclusion criteria were as follows: (i) births with missing child or parental national registration number (*n* = 76,649); (ii) births with missing data on gestational age (*n* = 1,126), maternal age (*n* = 13), or child sex (*n* = 7); and (iii) infants diagnosed with any major congenital malformations within the first year of life (*n* = 100,182). A total of 2,305,051 children meeting the eligibility criteria were included in the final analytic sample.

### Measures

#### Exposures

Intrauterine exposure to maternal diabetes served as the primary exposure, while paternal diabetes was used as a negative control exposure. Maternal diabetes was defined using the 10th revision of the International Classification of Disease (ICD-10) codes identified in the National Patient Register and the Medical Birth Register (Additional File 2: Table S1). The exposure information was categorized into three mutually exclusive groups: T1DM, T2DM, and GDM. T1DM and T2DM were identified based on ≥ 1 inpatient admission, or ≥ 2 outpatient visits (since 2001), or a diagnosis in the Medical Birth Register with an ICD code ever recorded before the index birth. The child’s birth date, rather than the conception date, was used to define T1DM and T2DM, as pre-existing but previously unidentified cases are likely to be detected during pregnancy [5]. GDM was identified in the current pregnancy, from date of conception until birth, using the following four criteria: (i) ≥ 1 inpatient, or (ii) ≥ 2 outpatient records in the National Patient Register, (iii) a diagnosis in the Medical Birth Register during pregnancy, and (iv) no diagnoses of pre-gestational T1DM or T2DM. A GDM diagnosis from a previous pregnancy was not carried forward to the index pregnancy unless a new diagnosis was confirmed. Paternal T1DM and T2DM were identified using ICD-10 codes E10 and E11, respectively. The positive predictive value (PPV) for diabetes in the National Patient Register exceeds 99% [30, 36], although its accuracy in distinguishing diabetes types requires further validation. In this study, mothers or fathers diagnosed with both T1DM and T2DM, possibly due to misclassification, were reclassified based on age at diabetes onset: T1DM if diagnosed before age 30 and T2DM if diagnosed after [37].

#### Study outcomes and follow-up

Epilepsy was ascertained based on the presence of at least one inpatient or two outpatient visits registered with the ICD-10 code G40 or G41. The healthcare registers have generally shown high validity in identifying epilepsy cases using the ICD codes [38, 39], with a PPV of approximately 90% reported in the National Patient Register [30, 40]. The primary outcome of interest was epilepsy of any type. However, we also studied focal (G400-G402) and generalized epilepsy (G403) separately, given their distinct etiology [24, 41]. Follow-up began at birth and ended at the earliest on the date of first epilepsy diagnosis, death, emigration, or October 31 st, 2023, whichever came first. The children who died (*n* = 6,099) or emigrated (*n* = 73,108) before receiving an epilepsy diagnosis were right-censored at the date of death or emigration (Additional File 3: Fig. S1). The Cause of Death Register provided data on death dates, while emigration dates were obtained from the Total Population Register.

#### Potential mediators

The mediators selected a priori were preeclampsia, preterm birth, birth asphyxia, and birthweight for gestational age [7–9, 42]. Preeclampsia was defined as gestational hypertension combined with proteinuria, using the ICD-10 codes O14 and O15. Preterm birth was defined as gestational age between 22 and 36 completed weeks. Gestational age was primarily assessed by ultrasound, and by the last menstrual period if data from ultrasound were not available. Birth asphyxia was defined as an Apgar score ≤ 3 at 5 min, or the presence of any of the following neonatal complications recorded within the first 27 days of life: convulsions, meconium aspiration, and hypoxic-ischemic encephalopathy (Additional File 2: Table S1) [43]. Birthweight was standardized for gestational age using Sweden’s national fetal growth reference curve [44]. Infants were then categorized by sex-specific birthweight percentiles into three groups: small for gestational age (SGA, below the 10th percentile), appropriate for gestational age (AGA, 10th to 90th percentile), and large for gestational age (LGA, above the 90th percentile).

#### Potential confounders

Confounder selection was based on subject-matter expertise and existing epidemiological literature on maternal diabetes and child neurodevelopment [15, 18, 42], focusing on factors that could influence the exposure-outcome, exposure-mediator, and mediator-outcome relationships [45]. These include infant characteristics, i.e., sex (male/female) and birth year (5-year intervals), and maternal characteristics i.e., maternal age at childbirth (< 20, 20–24, 25–29, 30–34, and ≥ 35 years), parity (1, 2, 3, ≥ 4), country of birth (Sweden, other Nordic and Non-Nordic country), cohabitation status, smoking in early pregnancy (no/yes), body mass index (BMI) in early pregnancy calculated as weight in kg/height in meter squared (underweight < 18.5, normal weight 18.5 to < 25, overweight 25 to < 30, and obesity ≥ 30), alcohol use disorder diagnosed in pregnancy (no/yes), chronic hypertension (no/yes), maternal history of epilepsy (no/yes), psychiatric disorders (no/yes), and use of insulin or oral antidiabetics in pregnancy (no/yes). The paternal covariates available were paternal age at childbirth (< 20, 20–24, 25–29, 30–34, and ≥ 35 years), paternal history of epilepsy (no/yes), and psychiatric disorders (no/yes).

The maternal characteristics were measured prior to or during the current pregnancy (Additional File 3: Fig. S1). However, when maternal height was missing in the current pregnancy, values from the most recent prior pregnancy were used as a proxy to define BMI. Maternal and paternal disease covariates were obtained from the National Patient Register and/or the Medical Birth Register, while data on maternal use of diabetes medications during pregnancy were obtained from the Prescription Drug Register (Additional File 2: Table S1). Mother’s country of birth and level of education were obtained from the Total Population Register and the Education Register, respectively. The Multi-generation Register was used to retrieve information on paternal age.

### Statistical analyses

All statistical analyses were performed in Stata (version 18.0) according to a prespecified analytical plan. We assessed the distribution of maternal and infant characteristics by maternal diabetes status. Crude incidence rates of epilepsy were calculated for each exposure category and were reported with 95% Confidence Intervals (CI). The Nelson-Aalen cumulative hazard function was plotted to compare the cumulative incidence of epilepsy among the children exposed to maternal diabetes versus those unexposed.

Hazard ratios (HR) were estimated using Cox proportional hazards models to examine the associations between maternal and paternal diabetes and epilepsy in children. The proportional hazards assumption was evaluated by log-minus-log survival curves, which were reasonably parallel (Additional File 3: Fig. S3) and by a global test of Schoenfeld residuals for the exposure categories (*p*-value 0.785), indicating no strong evidence against the assumption. Attained age was used as the underlying time scale in all Cox models. The HRs were adjusted for infant sex and birth year; maternal age at childbirth, parity, educational level, country of birth, cohabitation status, smoking, BMI, alcohol use disorders, chronic hypertension, history of epilepsy, and psychiatric disorders; as well as paternal age at childbirth, history of epilepsy, and psychiatric disorders. Maternal versus paternal T1DM and T2DM were mutually adjusted for each other, considering the high concordance of diabetes in couples through assortative mating and shared lifestyle [46]. Robust standard errors were estimated in all models to account for the clustering of siblings born to the same mother.

#### Negative control exposure analysis

We used paternal T1DM and T2DM as negative control exposures to evaluate uncontrolled confounding due to shared genetic or familial factors. A negative control exposure shares unobserved confounding with the primary exposure, but is not associated with the outcome through the hypothesized causal mechanism [47]. If genetics or shared family environment causes both maternal diabetes and child epilepsy [26], we would expect a similar association for paternal diabetes (Additional File 3: Fig. S2). However, since fathers do not provide the intrauterine environment, any association observed with paternal diabetes would suggest that the maternal association may be confounded [48, 49].

#### Sequential mediation analysis

In an initial investigation, we assessed the exposure-mediator and mediator-outcome associations (Additional File 2: Table S3 and Table S4). Maternal T1DM and T2DM were associated with higher odds of preeclampsia, preterm birth, and birth asphyxia (Additional File 2: Table S3), each of which, in turn, was associated with an increased risk of epilepsy. However, maternal T1DM and T2DM were not associated with increased risk of SGA, and LGA was not associated with epilepsy, therefore both were excluded from the final mediation analysis.

**Fig. 1:**
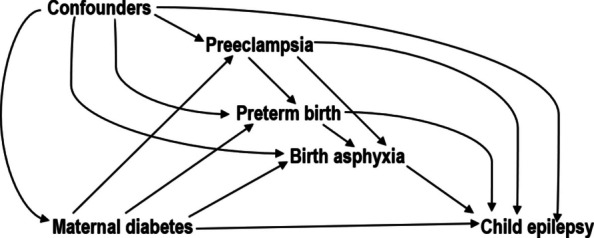
Illustration of the causal mediation framework of the study. Measured confounders include maternal socio-demographics (age, parity, country of birth, cohabitation status, educational level), maternal health (BMI, smoking, medication use, alcohol use disorder, chronic hypertension, psychiatric disorders, history of epilepsy), paternal health (diabetes, psychiatric disorders, epilepsy history, age), and child socio-demographics (age, sex, birth year). Unmeasured confounders may include genetics not captured by family history of epilepsy, maternal diet, physical inactivity, excessive gestational weight gain, and placental pathologies that may influence the mediators-outcome pathways

We used the difference-in-coefficients method [50] to partition the total effect of maternal T1DM and T2DM on children’s epilepsy into the indirect effect (i.e., the effect mediated through preeclampsia, preterm birth and birth asphyxia) and the direct effect (i.e., the effect that involves other mechanisms). A sequential mediation approach [51] was used to disentangle the mediator-specific pathways, assuming a causal order: T1DM/T2DM → Preeclampsia → Preterm birth → Birth asphyxia → Epilepsy (Fig. 1). This relied on the assumptions that there were no unmeasured confounders affecting the exposure-outcome, exposure-mediator, or mediator-outcome associations and no unmeasured mediator-outcome confounders affected by the exposure [45]. The difference-in-coefficients method further assumes that there are no interactions between the exposure and the mediators. This assumption held, as no evidence of interaction was observed when exposure-mediator interaction terms were included in the model.

We quantified multiple indirect pathways through the individual mediators and their combinations: (i) mediation through preeclampsia (and its causal descendants), (ii) joint mediation through preeclampsia and preterm birth, and (iii) joint mediation through all mediators: preeclampsia, preterm birth, and birth asphyxia. We then estimated: (iv) preterm birth’s contribution independent of preeclampsia by calculating the difference between (ii) and (i); (v) the joint contribution of preterm birth and birth asphyxia independent of preeclampsia by calculating the difference between (iii) and (i); and (vi) birth asphyxia’s contribution independent of preterm birth and preeclampsia by calculating the difference between (iii) and (ii). The 95% CIs for all mediation parameters were obtained through bootstrapping, with 1000 replications. We implemented the sequential mediation analysis using our author-written Stata program, which is provided in the Supplement (Additional File 1: Text S2).

#### Missing data analysis

The total proportion of missing data across all analytic variables was 11.3%, with the highest missing for maternal BMI. To deal with missing data, multiple imputations by chained equations method was used to generate 12 imputed datasets [52]. We imputed missing data under the missing-at-random assumption. The assumption’s plausibility was assessed by identifying predictors of missingness (Additional File 2: Table S2). The imputation model included all the analytic variables and the Nelson-Aalen cumulative hazard function.

#### Sensitivity analyses

We conducted a series of sensitivity analyses to evaluate the robustness of the main results. First, because our primary cohort by definition included live-born children who are selectively healthier than stillbirths [53, 54], we performed additional analysis including both stillbirths (*n* = 8054) and live births. In this analysis, we defined a composite outcome comprising stillbirths, postnatal deaths, and epilepsy and applied the Fine and Gray model [55] to estimate subdistribution HRs for epilepsy. Stillbirths and postnatal deaths were treated as competing events that preclude an epilepsy diagnosis. Follow-up began at the 22 weeks of gestation, the earliest point at which stillbirths were formally registered. Second, to evaluate robustness of the study findings to unmeasured confounding, we calculated E-values [56] for the total, direct, and indirect effects of maternal T1DM and T2DM as well as for the associations between each mediator and the outcome. Third, while preterm birth was a binary mediator, we have conducted an additional mediation analysis with gestational weeks (range 22–46) modelled as a continuous mediator, using the restricted cubic splines to allow for a non-linear association. Fourth, we fitted restricted cubic splines for the continuous covariates (i.e., child’s birth year and maternal age, parity, years of education, and BMI) in the adjusted Cox model to account for nonlinearity. Fifth, we examined if child’s sex modified the association between maternal diabetes and epilepsy. Sixth, since diabetes and epilepsy are comorbid disorders [27], we excluded all children with diabetes (*n* = 13,490) and all parents with a history of epilepsy (*n* = 24,382) to account for potential shared genetic susceptibility. Seventh, to better distinguish the overlapping diagnoses of maternal T1DM and T2DM, we used prescription data on insulin available since 2005. Mothers with diabetes onset at age ≤ 30 and treated with insulin only were classified as having T1DM. We also repeated the analysis excluding mothers with overlapping diagnostic codes. Eighth, the associations were re-examined by restricting the definition of maternal T1DM and T2DM to the preconception period. Ninth, we investigated whether maternal use of insulin or oral antidiabetic drugs played any mediating or moderating role, restricting the analysis to children born to mothers with diabetes in 2005 or later. Tenth, we assessed the impact of diabetes duration by categorizing maternal T1DM and T2DM according to the time since diagnosis (< 5 years, 5–10 years, or > 10 years before childbirth). Eleventh, to account for potential ethnic heterogeneity [57], a subgroup analysis was conducted among women born in Sweden only. Twelfth, we restricted the analysis to children born before 2018 due to a recent change in GDM diagnostic criteria in Sweden [58]. Thirteenth, we examined whether the severity of GDM, assessed by the need for antidiabetic medication treatment [59], increases the risk of child epilepsy. Fourteenth, a sensitivity analysis was also conducted, including infants born with major congenital malformations.

## Results

Of the 2,305,051 children included in the analysis (female 48.9%), 14,283 (0.6%) were exposed to maternal pre-gestational T1DM, 3833 (0.2%) to T2DM, and 36,388 (1.6%) to GDM. Compared with mothers without diabetes, those diagnosed with T2DM or GDM were more likely to be older (> 34 years), live alone, be overweight or obese, have higher parity (≥ 4 children) and lower education. Mothers with diabetes also had a higher prevalence of hypertension, epilepsy, psychiatric disorders, alcohol use disorders, and preeclampsia. In comparison with T1DM mothers, T2DM mothers were more likely to be older, multiparous, born outside Sweden, and had lower education and higher prevalence of smoking, obesity, and psychiatric disorders. Children of mothers with diabetes were more likely to be born preterm or LGA and have asphyxia-related complications at birth. Most mothers with T2DM used diabetes medications (i.e., insulin or oral antidiabetics) during pregnancy, whereas a smaller proportion GDM received such treatment (Table 1).

**Table 1:** Maternal and infant characteristics according to maternal pre-gestational and gestational diabetes status

**Sample characteristics**	**Total** (*N* = 2,305,051)	**Unexposed** (*N* = 2,250,547)	**Exposed to maternal diabetes mellitus**		
			**T1DM** (*N* = 14,283)	**T2DM** (*N* = 3,833)	**GDM** **(***N* = 36,388)
**Infant characteristics** % (N)					
Sex					
Male	51.1 (1,177,861)	51.1 (1,149,950)	51.0 (7,280)	50.0 (1,918)	51.4 (18,713)
Female	48.9 (1,127,190)	48.9 (1,100,597)	49.0 (7,003)	50.0 (1,915)	48.6 (17,675)
Birth year					
1998–2002	17.9 (412,722)	18.1 (406,762)	13.9 (1,991)	5.7 (217)	10.3 (3,752)
2003–2007	20.2 (464,794)	20.3 (457,258)	19.4 (2,777)	12.3 (471)	11.8 (4,288)
2008–2012	21.8 (503,103)	21.9 (493,683)	22.3 (3,183)	19.7 (757)	15.1 (5,480)
2013–2017	22.4 (516,445)	22.5 (505,385)	23.9 (3,415)	29.2 (1,119)	17.9 (6,526)
2018–2021	17.7 (407,987)	17.2 (387,459)	20.4 (2,917)	33.1 (1,269)	44.9 (16,342)
Preterm birth					
No	95.6 (2,202,806)	95.7 (2,154,201)	79.9 (11,412)	88.2 (3,381)	92.9 (33,812)
Yes	4.4 (102,245)	4.3 (96,346)	20.1 (2,871)	11.8 (452)	7.1 (2,576)
Birth-asphyxia^a^					
No	99.5 (2,293,517)	99.5 (2,239,511)	98.6 (14,077)	98.6 (3,780)	99.3 (36,149)
Yes	0.5 (11,534)	0.5 (11,036)	1.4 (206)	1.4 (53)	0.7 (239)
Birthweight for gestational age					
Small for gestational age (SGA)	6.2 (142,200)	6.2 (139,679)	2.7 (390)	5.7 (220)	5.3 (1,911)
Appropriate for gestational age (AGA)	81.6 (1,879,781)	82.0 (1,845,949)	43.1 (6,153)	62.3 (2,388)	69.5 (25,291)
Large for gestational age (LGA)	12.1 (277,919)	11.6 (260,430)	51.2 (7,306)	30.8 (1,181)	24.7 (9,002)
Missing	0.2 (5,151)	0.2 (4,489)	3.0 (434)	1.1 (44)	0.5 (184)
**Maternal characteristics** % (N)					
Age at childbirth (years)					
≤ 19	1.3 (30,403)	1.3 (30,074)	1.0 (142)	0.2 (8)	0.5 (179)
20–24	12.1 (277,968)	12.2 (273,648)	10.7 (1,527)	4.5 (174)	7.2 (2,619)
25–29	31.1 (716,257)	31.2 (702,379)	31.6 (4,512)	14.7 (565)	24.2 (8,801)
30–34	35.0 (805,797)	35.0 (787,137)	34.0 (4,855)	29.9 (1,147)	34.8 (12,658)
≥ 35	20.6 (474,626)	20.3 (457,309)	22.7 (3,247)	50.6 (1,939)	33.3 (12,131)
Parity					
1	43.4 (1,000,628)	43.6 (980,437)	42.7 (6,104)	25.7 (984)	36.0 (13,103)
2	37.3 (860,726)	37.4 (841,709)	36.8 (5,263)	31.8 (1,217)	34.5 (12,537)
3	13.5 (310,749)	13.4 (301,844)	13.6 (1,942)	20.1 (772)	17.0 (6,191)
≥ 4	5.8 (132,948)	5.6 (126,557)	6.8 (974)	22.4 (860)	12.5 (4,557)
Country of birth					
Sweden	76.7 (1,768,112)	77.1 (1,734,445)	84.3 (12,039)	47.7 (1,829)	54.4 (19,799)
Other Nordic country	1.5 (35,348)	1.5 (34,654)	1.2 (171)	1.4 (54)	1.3 (469)
Non-Nordic country	21.7 (499,608)	21.3 (479,506)	14.4 (2,063)	50.8 (1,946)	44.2 (16,093)
Missing	0.1 (1,983)	0.1 (1,942)	0.1 (10)	0.1 (4)	0.1 (27)
Level of education (years)					
≤ 9	8.3 (190,343)	8.1 (182,972)	10.0 (1,427)	20.6 (788)	14.2 (5,156)
10–11	11.1 (254,716)	11.0 (247,355)	11.8 (1,683)	17.5 (672)	13.8 (5,006)
12	25.8 (594,172)	25.8 (579,968)	28.6 (4,081)	23.8 (914)	25.3 (9,209)
13–14	14.5 (334,141)	14.5 (326,498)	13.5 (1,928)	12.8 (490)	14.4 (5,225)
≥ 15	39.0 (898,838)	39.2 (882,764)	35.4 (5,054)	20.8 (799)	28.1 (10,221)
Missing	1.4 (32,841)	1.4 (30,990)	0.8 (110)	4.4 (170)	4.3 (1,571)
Cohabitation with partner					
No	5.0 (114,985)	5.0 (111,681)	5.6 (800)	8.4 (322)	6.0 (2,182)
Yes	90.2 (2,078,116)	90.2 (2,029,274)	88.9 (12,703)	86.9 (3,329)	90.2 (32,810)
Missing	4.9 (111,950)	4.9 (109,592)	5.5 (780)	4.7 (182)	3.8 (1,396)
Smoking in early pregnancy					
No	89.1 (2,053,286)	89.1 (2,004,399)	87.1 (12,447)	87.9 (3,368)	90.9 (33,072)
Yes	7.2 (166,289)	7.2 (162,277)	8.0 (1,139)	9.4 (359)	6.9 (2,514)
Missing	3.7 (85,476)	3.7 (83,871)	4.9 (697)	2.8 (106)	2.2 (802)
BMI in early pregnancy (kg/m^2^)					
Underweight (≤ 18.4)	2.2 (51,828)	2.3 (51,402)	0.6 (88)	0.6 (23)	0.9 (315)
Normal weight (18.5–24.9)	54.7 (1,261,814)	55.4 (1,246,313)	39.4 (5,622)	15.8 (607)	25.5 (9,272)
Overweight (25–29.9)	23.5 (541,482)	23.4 (525,881)	30.7 (4,385)	26.6 (1,019)	28.0 (10,197)
Obese (≥ 30)	11.6 (266,415)	11.0 (246,601)	21.5 (3,074)	51.3 (1,967)	40.6 (14,773)
Missing	8.0 (183,512)	8.0 (180,350)	7.8 (1,114)	5.7 (217)	5.0 (1,831)
Alcohol use disorders in pregnancy					
No	98.6 (2,272,439)	98.6 (2,219,430)	96.7 (13,812)	95.8 (3,670)	97.6 (35,527)
Yes	1.4 (32,612)	1.4 (31,117)	3.3 (471)	4.2 (163)	2.4 (861)
Epilepsy					
No	99.4 (2,291,840)	99.4 (2,237,799)	98.9 (14,120)	99.0 (3,793)	99.3 (36,128)
Yes	0.6 (13,211)	0.6 (12,748)	1.1 (163)	1.0 (40)	0.7 (260)
Psychiatric disorders					
No	90.9 (2,094,341)	91.0 (2,047,847)	83.6 (11,946)	81.0 (3,106)	86.4 (31,442)
Yes	9.1 (210,710)	9.0 (202,700)	16.4 (2,337)	19.0 (727)	13.6 (4,946)
Chronic hypertension					
No	99.3 (2,289,156)	99.4 (2,236,260)	96.0 (13,708)	93.1 (3,570)	97.9 (35,618)
Yes	0.7 (15,895)	0.6 (14,287)	4.0 (575)	6.9 (263)	2.1 (770)
Preeclampsia					
No	97.3 (2,241,775)	97.4 (2,191,331)	86.6 (12,368)	92.9 (3,562)	94.8 (34,514)
Yes	2.7 (63,276)	2.6 (59,216)	13.4 (1,915)	7.1 (271)	5.2 (1,874)
Insulin use in pregnancy^b^					
No	99.0 (1,693,357)	100.0 (1,665,174)	11.0 (1,236)	38.0 (1,310)	81.9 (25,286)
Yes	1.0 (17,510)	0.01 (129)	89.0 (10,014)	62.0 (2,139)	18.1 (5,579)
Oral antidiabetics use in pregnancy^b^					
No	99.6 (1,703,561)	99.9 (1,663,978)	94.6 (10,641)	61.6 (2,125)	86.9 (26,817)
Yes	0.4 (7,306)	0.1 (1,325)	5.4 (609)	38.4 (1,324)	13.1 (4,048)

*BMI* body mass index, *GDM* gestational diabetes mellitus, *T1DM* type 1 diabetes mellitus, *T2DM* type 1 diabetes mellitus

^a^Birth asphyxia was defined as any of the following complications: low Apgar score, convulsions, meconium aspiration, and hypoxic-ischemic encephalopathy

^b^Information on prescription drugs for diabetes was available from July 2005 onward (*N* = 1,710,867)

**Fig. 2:**
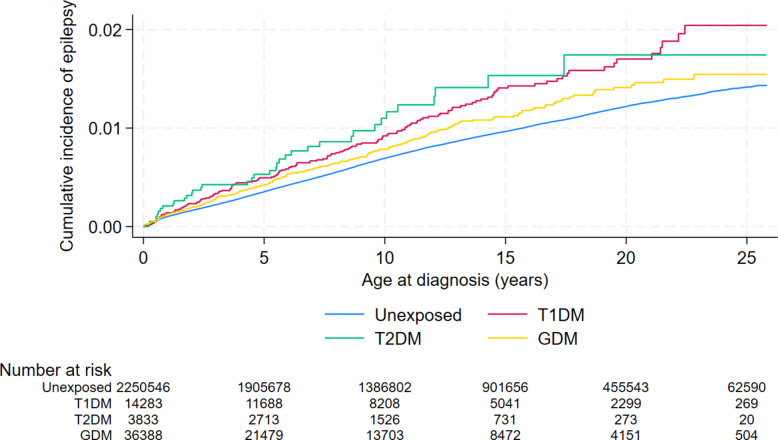
Unadjusted cumulative incidence of epilepsy among children exposed and unexposed to maternal diabetes. Abbreviations: GDM, Gestational diabetes mellitus; TIDM, Type 1 diabetes mellitus; T2DM, Type 2 diabetes mellitus

Over a follow-up period of up to 25.8 years (median: 13 years, interquartile range: 7 to 19 years), 18,968 children developed epilepsy, corresponding to an incidence rate of 63.7 (95% CI: 62.8, 64.6) per 100,000 person-years. The majority of epilepsy cases (71%) were diagnosed by 10 years of age (Additional File 3: Fig. S4). The incidence rates of focal epilepsy and generalized epilepsy were 23.8 (*n* = 7,156) and 15.2 (*n* = 4,564) per 100,000 person-years, respectively. Unadjusted cumulative incidence curves indicated a higher hazard of epilepsy among children of mothers with T1DM (89.8 per 100,000 person-years), T2DM (102.9 per 100,000 person-years), and GDM (76.2 per 100,000 person-years) compared with those without these conditions (63.3 per 100,000 person-years) throughout the follow-up period (Fig. 2, Table 2).

**Table 2:** Incidence rate of epilepsy in children by maternal and paternal diabetes status

**Exposure/s**	**No. of children**	**No. of epilepsy**	**Rates (95% CI)**^a^
**Maternal diabetes**			
Unexposed	2,250,547	18,518	63.3 (62.4, 64.2)
T1DM	14,283	156	89.8 (76.8, 105.1)
T2DM	3833	37	102.9 (74.5, 142.0)
GDM	36,388	257	76.2 (67.4, 86.1)
**Paternal diabetes**			
Unexposed	2,286,867	18,830	63.6 (62.7, 64.6)
T1DM	13,017	100	67.2 (55.2, 81.7)
T2DM	5167	38	68.9 (50.1, 94.7)

*CI* confidence interval, *GDM* Gestational diabetes mellitus, *T1DM* type 1 diabetes mellitus, *T2DM* type 2 diabetes mellitus

^a^Crude incidence rates per 100,000 person-years

**Fig. 3:**
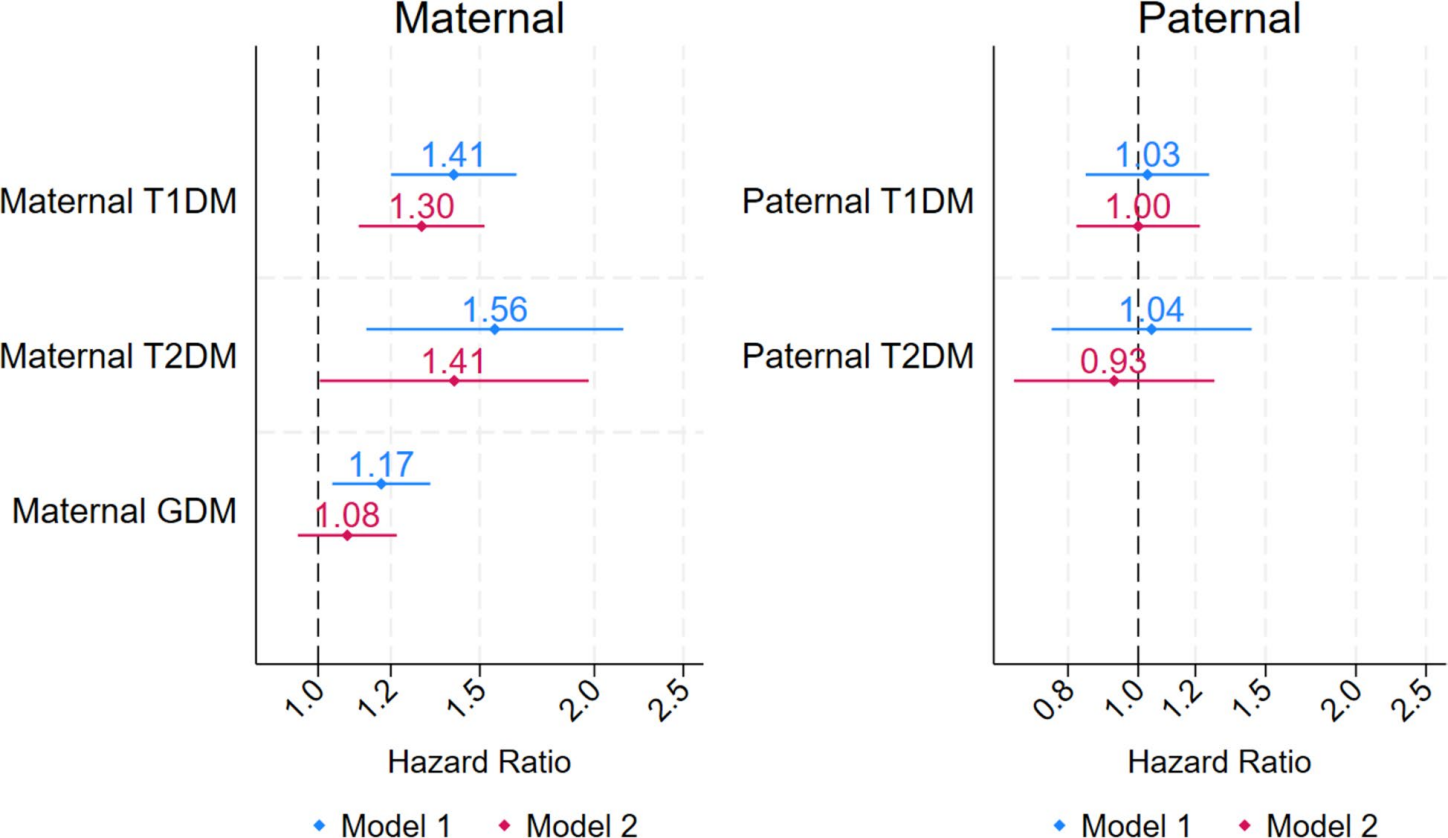
Hazard ratios (with 95% CI) of the associations between maternal and paternal diabetes status and epilepsy in children. The HRs were pooled from 12 multiply imputed datasets. Model 1 shows the unadjusted HRs. Model 2 was adjusted for infant sex and birth year; maternal age at childbirth, parity, country of birth, educational level, cohabitation status, smoking, body mass index, alcohol use disorders, chronic hypertension, history of epilepsy, and psychiatric disorders; and paternal age at childbirth, history of epilepsy, and psychiatric disorders. Maternal and paternal diabetes were mutually adjusted for each other.. Abbreviations: GDM, Gestational diabetes mellitus; T1DM, type 1 diabetes mellitus; T2DM, type 2 diabetes mellitus

In the adjusted Cox regression model (Fig. 3), maternal T1DM was associated with a 30% higher rate of epilepsy (adjusted HR: 1.30; 95% CI: 1.11, 1.52) compared with no exposure to maternal diabetes. T2DM was associated with a 41% higher rate (adjusted HR: 1.41; 95% CI: 1.01, 1.97). No association was found between GDM and epilepsy in children (adjusted HR: 1.08; 95% CI: 0.95, 1.22). Paternal T1DM (adjusted HR: 1.00; 95% CI: 0.82, 1.22) and T2DM (adjusted HR: 0.93; 95% CI: 0.67, 1.27) were not associated with an increased HR of epilepsy (Fig. 2). When the two subtypes of child epilepsy were analysed separately (Table 3), maternal T1DM was associated with an increased risk of generalized epilepsy (adjusted HR: 1.53; 95% CI: 1.13, 2.06), but not with focal epilepsy. Maternal T2DM was associated with elevated HRs for both epilepsy subtypes; however, these associations were not statistically significant.

**Table 3:** Incidence rates and hazard ratios of focal and generalized epilepsy in children by maternal and paternal diabetes status

Exposure/s	Outcome: Focal epilepsy in children			
	No. of cases	Rates^a^	Model 1 HR (95% CI)	Model 2 HR (95% CI)
**Maternal diabetes**				
Unexposed	6995	23.8	1.00 (Ref.)	1.00 (Ref.)
T1DM	51	29.2	1.22 (0.92, 1.60)	1.15 (0.88, 1.52)
T2DM	15	41.5	1.71 (1.03, 2.84)	1.58 (0.95, 2.62)
GDM	95	28.1	1.20 (0.98, 1.46)	1.15 (0.94, 1.40)
**Paternal diabetes**				
Unexposed	7108	23.9	1.00 (Ref.)	1.00 (Ref.)
T1DM	34	22.8	0.93 (0.66, 1.30)	0.92 (0.66, 1.29)
T2DM	14	25.3	1.02 (0.60, 1.72)	0.96 (0.57, 1.62)
	**Outcome: Generalized epilepsy in children**			
**Maternal diabetes**				
Unexposed	4454	15.2	1.00 (Ref.)	1.00 (Ref.)
T1DM	44	25.2	1.67 (1.24, 2.25)	1.53 (1.13, 2.06)
T2DM	10	27.6	1.89 (1.01, 3.51)	1.66 (0.89, 3.09)
GDM	56	16.5	1.12 (0.86, 1.46)	1.04 (0.80, 1.36)
**Paternal diabetes**				
Unexposed	4531	15.2	1.00 (Ref.)	1.00 (Ref.)
T1DM	26	17.4	1.15 (0.78, 1.69)	1.15 (0.78, 1.69)
T2DM	< 10^b^	-	-	-

*CI* confidence interval, *GDM* Gestational diabetes mellitus, *HR* hazard ratio, *T1DM* type 1 diabetes mellitus, *T2DM* type 2 diabetes mellitus

^a^Crude incidence rates per 100,000 person-years

^b^Exact count is suppressed for confidentiality reason. -Not estimated due to insufficient number of events

The HRs were pooled from 12 multiply imputed datasets. Model 1 shows the unadjusted HRs. Model 2 was adjusted for infant sex and birth year; maternal age at childbirth, parity, country of birth, educational level, cohabitation status, smoking, body mass index, alcohol use disorders, chronic hypertension, history of epilepsy and psychiatric disorders; and paternal age at childbirth, history of epilepsy, and psychiatric disorders. Maternal and paternal diabetes were mutually adjusted for each other

Table 4 presents the joint and mediator-specific effects of preeclampsia, preterm birth, and birth asphyxia on the associations between maternal T1DM and T2DM and epilepsy in children. Preeclampsia mediated 10.6% of the estimated total effect of maternal T1DM on epilepsy (adjusted HR^Indirect Effect^:1.03; 95% CI: 1.02, 1.04) while preeclampsia and preterm birth together mediated 35.9% (adjusted HR^Indirect Effect^:1.10; 95% CI: 1.08, 1.12). Thus, preterm birth alone mediated 25.3% of the effect independent of preeclampsia. Similarly, preterm birth and birth asphyxia, regardless of preeclampsia, jointly mediated nearly half of the effect (adjusted HR^Indirect Effect^:1.14, 95% CI: 1.11, 1.17). For the total effect of T2DM, 44.5% was mediated jointly by preeclampsia, preterm birth, and birth asphyxia, with birth asphyxia alone mediating 20.5%. After accounting for these mediators, the direct effects of maternal T1DM and T2DM were attenuated compared to the total effects but remained elevated, although not statistically significant (Table 4).

**Table 4:** Sequential mediation of the associations of maternal T1DM and T2DM with child epilepsy via preeclampsia, preterm birth, and birth asphyxia

Mediation parameters	(i) Mediation by preeclampsia	
	**T1DM** HR (95% CI)	**T2DM** HR (95% CI)
Total effect	1.30 (1.10, 1.52)	1.40 (1.02, 1.97)
Direct effect	1.26 (1.07, 1.49)	1.33 (0.95, 1.86)
Indirect effect	1.03 (1.02, 1.04)	1.05 (1.04, 1.07)
Proportion mediated (%)	10.6	14.7
	**(ii) Joint mediation by preeclampsia and preterm birth**	
	**T1DM** HR (95% CI)	**T2DM** HR (95% CI)
Total effect	1.30 (1.10, 1.52)	1.40 (1.02, 1.97)
Direct effect	1.18 (1.01, 1.38)	1.29 (0.92, 1.80)
Indirect effect	1.10 (1.08, 1.12)	1.08 (1.06, 1.10)
Proportion mediated (%)	35.9	24.1
	**(iii) Joint mediation by preeclampsia, preterm birth and birth asphyxia**	
	**T1DM** HR (95% CI)	**T2DM** HR (95% CI)
Total effect	1.30 (1.10, 1.52)	1.40 (1.01, 1.96)
Direct effect	1.12 (0.95, 1.32)	1.21 (0.86, 1.68)
Indirect effect	1.17 (1.13, 1.20)	1.16 (1.09, 1.24)
Proportion mediated (%)	59.3	44.5
	**(iv) Mediation by preterm birth alone**	
	**T1DM** HR (95% CI)	**T2DM** HR (95% CI)
Indirect effect	1.07 (1.06, 1.08)	1.03 (1.02, 1.04)
Proportion mediated (%)	25.3	9.4
	**(v) Joint mediation by preterm birth and birth asphyxia**	
	**T1DM** HR (95% CI)	**T2DM** HR (95% CI)
Indirect effect	1.14 (1.11, 1.17)	1.11 (1.04, 1.18)
Proportion mediated (%)	48.7	29.9
	**(vi) Mediation by birth asphyxia alone**	
	**T1DM** HR (95% CI)	**T2DM** HR (95% CI)
Indirect effect	1.07 (1.04, 1.09)	1.07 (1.01, 1.14)
Proportion mediated (%)	23.4	20.5

*CI* confidence interval, *HR* hazard ratio, *T1DM* type 1 diabetes mellitus, *T2DM* type 2 diabetes mellitus

The 95% CIs are percentile based, obtained by bootstrapping with 1000 replications

The Proportion Mediated is a ratio of the Indirect and Total Effects (i.e., Indirect Effect/Total Effect), calculated on the log HR scale and expressed in percentage

T1DM and T2DM were used as binary exposures (No/Yes) in the mediation analysis

The HRs were adjusted for infant sex and birth year; maternal age at childbirth, parity, country of birth, educational level, cohabitation status, smoking, body mass index, alcohol use disorder, chronic hypertension, history of epilepsy, and any psychiatric disorders; and paternal age at childbirth, paternal T1DM or T2DM, history of epilepsy, and psychiatric disorders

### Sensitivity analyses

Results from the sensitivity analyses are presented below: First, in the competing risk analysis (Additional File 2: Table S5), which included both live births and stillbirths, the subdistribution HRs for maternal T1DM and T2DM were similar to the HRs in the restricted live-born cohort. Second, the E-values for the Total Effect of maternal T1DM and T2DM on epilepsy were much greater in magnitude than the observed estimates. While the E-values for the Indirect Effects via preterm birth and birth asphyxia were relatively small, the E-values for the mediators-outcome associations were high (Additional File 2: Table S6), suggesting that the causal mediation parameters were robust to unmeasured confounding. Third, when modelled as a continuous variable with a spline function, gestational age tended to mediate a larger proportion of the associations than when preterm birth was modelled as a binary mediator (Additional File 2: Table S7).

Fourth, using restricted cubic splines for continuous covariates led to a slight attenuation of the association between maternal T2DM and child epilepsy, although the association for maternal T1DM remained unchanged (Additional File 2: Table S8). Fifth, the HRs for the associations between maternal diabetes subtypes and child epilepsy were not significantly different between males and females (interaction *p*-value: 0.617; Additional File 2: Table S9). Sixth, the associations were robust to the exclusion of children with diabetes or those with a parental history of epilepsy (Additional File 2: Table S10). Seventh, reclassifying overlapping maternal diagnoses of T1DM and T2DM based on insulin use led to minimal changes in the HR estimates (Additional File 2: Table S11), but excluding mothers diagnosed with both T1DM and T2DM resulted in elevated estimates (Additional File 2: Table S12). Eight, when maternal T1DM and T2DM diagnoses were restricted to the preconception period, the HR for T1DM was similar, whereas the T2DM cases dropped, resulting in imprecise estimates (Additional File 2: Table S13).

Ninth, maternal use of insulin or oral antidiabetic medications during pregnancy was not associated with epilepsy risk in children (Additional File 2: Table S14), and no evidence of effect modification by maternal medication use was observed (interaction *p*-value: 0.731). Tenth, the HRs for maternal T1DM diagnosed within 5 years preceding childbirth did not differ significantly from those diagnosed earlier, as indicated by the overlapping 95% CIs (Additional File 2: Table S15). Eleventh, results were unchanged when restricting the analysis to Swedish-born mothers (Additional File 2: Table S16). Twelfth, the GDM estimate was closer to null in the analysis restricted to children born before 2018, prior to the new diagnostic criteria (Additional File 2: Table S17). Thirteenth, GDM requiring diabetes medications was not associated with child epilepsy after adjustment for potential confounders (Additional File 2: Table S18). Fourteenth, inclusion of infants with congenital malformations led to a modest increase in the HR for maternal T1DM and attenuation for T2DM (Additional File 2: Table S19).

## Discussion

### Main findings

This national cohort study in Sweden suggests that children exposed in utero to maternal pre-existing T1DM or T2DM had an increased risk of developing epilepsy during childhood and early adulthood. Preterm birth and asphyxia-related neonatal complications partially mediated these associations. We observed no association between GDM and epilepsy in children. Paternal T1DM and T2DM were not associated with an elevated risk of epilepsy.

### Comparison with the literature and interpretations

Given the scarce evidence on maternal diabetes and childhood epilepsy, direct comparison with prior studies is limited. However, our study findings partly align with the population-based study in Taiwan [20], which followed children up to age 12 and found a higher incidence of epilepsy and other neurodevelopmental disorders among those born to mothers with T1DM or T2DM, but not with GDM. In contrast to our results, the authors reported larger effect sizes (HR for T1DM: 2.33; HR for T2DM: 1.49) and lower T1DM prevalence (0.04%), raising concerns about statistical power and confounding bias. Notably, our nationwide study benefited from comprehensive registries and confounding control, whereas the Taiwanese study did not adjust for key factors, including maternal history of epilepsy, psychiatric disorders, obesity, and parity, thereby limiting the interpretability of its results.

Our findings add to the growing body of research linking maternal diabetes, particularly pre-gestational diabetes, to various childhood neurodevelopmental disorders, such as ADHD, ASD, intellectual disability, and cerebral palsy [11, 14, 16, 18]. Reported HRs typically range from 1.15 to 1.6 [11, 14, 16], comparable to our epilepsy estimates. Similarly, a systematic review and meta-analysis of 202 observational studies (56.1 million pregnancies) found that exposure to maternal diabetes raised the risk of any neurodevelopmental disorder in offspring by 1.28-fold [13].

The current study further extends the past literature by differentiating between focal and generalized epilepsy. While localized brain injuries may lead to focal epilepsy, generalized epilepsy may result from brain-wide altered neural networks and was shown to be associated with poorer school performance than focal epilepsy [41]. We found that maternal T1DM was specifically associated with generalized epilepsy, while GDM and paternal T1DM were not, indicating a role of chronic early intrauterine metabolic disturbances in brain development. Maternal T2DM may also increase the risk of generalized or focal epilepsy, but estimates were imprecise due to the few exposed cases.

T1DM is an autoimmune disease, whereas T2DM is characterized by chronic insulin resistance; both can lead to chronic hyperglycaemia. In the current study, maternal T1DM and T2DM were associated with similar increases in children’s overall risk of epilepsy, implying a shared pathological mechanism. Also, null associations between paternal T1DM or T2DM and child epilepsy [60] in this study make a genetic explanation less likely. Thus, the increased epilepsy risk in children prenatally exposed to maternal pre-gestational diabetes may mainly result from hyperglycaemia or other diabetes-related physiological alterations during key periods of fetal development [6, 12, 61, 62].

Hyperglycaemia can alter placental structure and function [63] and increase preeclampsia risk [64, 65], which may lead to growth restriction, preterm delivery, and birth asphyxia [66]. In our study, preterm birth and birth asphyxia, also common in pre-gestational diabetes without preeclampsia [67], each mediated about one-quarter of the total effect of T1DM on child epilepsy. This supports prior evidence that obstetric and neonatal complications may substantially mediate the relationship between maternal pre-gestational diabetes and child neurodevelopmental outcomes [15, 42]. Consistent with this, randomized controlled trials have shown that early intervention programs for infants born preterm or with asphyxia can improve neurocognitive and motor outcomes [68–70] and potentially reduce the incidence of childhood epilepsy [71].

The lack of an association between maternal GDM and epilepsy suggests that the timing, level, and duration of hyperglycaemic exposure during pregnancy are important [72]. Unlike GDM, typically diagnosed in the second or third trimester, T1DM and T2DM expose the fetus to hyperglycaemia already from conception, with potential negative impact on brain development leading to epilepsy [14, 21]. A meta-analysis found that early-diagnosed GDM was associated with elevated risks of ADHD and ASD, while late diagnosis showed no significant association [13]. Given possible misclassification of GDM timing [73], more precise data are needed to clarify its role in child neurodevelopmental disorders, including epilepsy.

### Clinical implications

Considering the rising prevalence of diabetes in women of reproductive age [74], the study findings underscore the need for optimal management of maternal diabetes and related birth complications to reduce epilepsy risk. Strengthening interventions to prevent preterm births and birth asphyxia, along with postnatal management strategies for at-risk infants, may partly help prevent epilepsy linked to maternal diabetes. For example, therapeutic hypothermia (cooling) is an established intervention for infants born with asphyxia, likely protecting the central nervous system and improving neurological outcomes [70, 71]. However, maternal diabetes itself remains a modifiable and potentially more impactful intervention target, given its possible direct effects through unexplored pathways. Timely management of maternal hyperglycaemia, particularly before and during early pregnancy, may benefit long-term child neurodevelopment. Emerging technologies, such as automated insulin delivery pumps with pregnancy-specific algorithms, may further optimize glycaemic control [75]. Future research should track time in range and glucose variability across pregnancy to find potential critical windows of vulnerability.

### Strengths and limitations

The strengths of this study include the use of a large, nationwide sample, which enabled separate analysis of maternal T1DM and T2DM in relation to the rare outcome of epilepsy, including its focal and generalized subtypes that have been infrequently examined in previous research. Leveraging the comprehensive Swedish health registers, the study achieved complete follow-up of children for up to nearly age 26, thereby minimizing the risk of selection or recall bias and allowing adjustment for a robust set of confounders. The availability of data on paternal diabetes facilitated a family-based design to assess unmeasured familial confounding. The sequential mediation approach, by design, partly addressed exposure-induced intermediate confounding, in which an upstream mediator may also act as a confounder [45].

However, the findings should be considered in light of several limitations. First, although we combined multiple data sources and required two or more ICD-coded outpatient records to ascertain diabetes cases, some degree of misclassification may remain. Differential misclassification of child epilepsy is also possible if mothers with diabetes seek medical care more often. Nonetheless, any potential misclassification bias is unlikely to substantially affect the results, given the high accuracy of chronic disease diagnoses in the National Patient Register [30, 76]. Second, despite comprehensive adjustment for confounders, residual confounding cannot be ruled out entirely, particularly for the mediation estimates. The E-value estimates from our sensitivity analysis indicate that the unmeasured confounding bias would have to be quite strong to fully explain away the observed associations. The E-value provides a single estimate for unmeasured mediator-outcome confounders but does not account for all sources of bias. More complex quantitative bias analyses would necessitate explicit assumptions about specific unmeasured confounders that are difficult to justify in registry-based data. The E-value should therefore be interpreted as an illustrative measure of robustness rather than a definitive assessment of bias. Third, if maternal diabetes increases the risk of fetal death, restricting the study population to live births may underestimate the association with childhood epilepsy [53, 54]. While our competing risk analysis incorporated stillbirth as a competing event to address this concern, selection bias related to early pregnancy loss cannot be entirely eliminated. Because follow-up in this analysis began earlier in pregnancy by design, immortal time bias may arise, particularly for GDM which is often diagnosed with some delay [73]. Fourth, despite the large overall sample, the small number of maternal T2DM cases limited statistical power for subgroup analyses. Finally, although the high coverage of Swedish registers enhances the generalizability of these findings to similar high-income countries, the relative ethnic homogeneity of the Swedish population may limit generalizability to more diverse settings.

## Conclusions

In this nationwide Swedish cohort study, pre-existing maternal T1DM and T2DM were associated with an increased risk of epilepsy in children. The negative-control analysis using paternal T1DM and T2DM, along with the sequential mediation analysis, supported an intrauterine origin for these associations. Preterm birth and asphyxia-related conditions at birth partially mediated the observed associations.

## Supplementary Information

Additional file 1. Supplementary Methods Text S1-Text S2. Text S1–Supplementary methods: description of data sources. Text S2–Author-written Stata program for sequential mediation analysis in a multiple, causally ordered mediators framework

Additional file 2. Supplementary Tables S1-S19. Table S1–International Classification of Disease-10 codes for maternal and child morbidity and the Anatomical Therapeutic Chemicalcodes for maternal drug use. Table S2–Distribution of sample characteristics according to missing and complete data. Table S3–Odds ratios for the associations between maternal diabetes and potential mediators. Table S4– Incidence rates and hazard ratios of epilepsy in children by potential mediating variables. Table S5–Incidence rates and subdistribution hazard ratios of epilepsy in children by maternal and paternal diabetes status, estimated through competing risk analysis including both live births and stillbirths. Table S6–E-values for the total, direct, and indirect effects of maternal T1DM and T2DM and for the mediator-outcome estimates. Table S7–Sequential mediation of the associations of maternal T1DM and T2DM with child epilepsy via preeclampsia, gestational age, and birth asphyxia. Table S8–Hazard ratios of epilepsy in children by maternal and paternal diabetes status, using the spline terms to fit continuous confounders. Table S9–Sex-stratified incidence rates and hazard ratios of epilepsy in children by maternal and paternal diabetes status. Table S10– Incidence rates and hazard ratios of epilepsy in children by maternal and paternal diabetes status, after excluding children with diabetes or parental epilepsy. Table S11–Incidence rates and hazard ratios of epilepsy in children by maternal diabetes status, reclassified based on insulin use. Table S12–Incidence rates and hazard ratios of epilepsy in children by maternal diabetes status, after excluding overlapping diagnoses of maternal T1DM and T2DM. Table S13–Incidence rates and hazard ratios of epilepsy in children by parental diabetes status, restricting maternal T1DM and T2DM diagnoses to the preconception period. Table S14–Incidence rates and hazard ratios of epilepsy in children by maternal use of diabetes medications during pregnancy. Table S15–Incidence rates and hazard ratios of epilepsy in children by duration of pre-gestational type 1 and type 2 diabetes. Table S16– Incidence rates and hazard ratios of epilepsy by maternal diabetes status among children of Swedish-born mothers. Table S17–Incidence rates and hazard ratios of epilepsy by maternal diabetes status before the revised GDM criteria in 2018. Table S18–Incidence rates and hazard ratios of epilepsy by severity of maternal GDM. Table S19–Incidence rates and hazard ratios of epilepsy in children by maternal and paternal diabetes status, with major malformations included

Additional file 3. Supplementary Fig. S1-S4. Fig. S1–Study timeline from exposure assessment to outcome follow up. Fig. S2–Illustration of the negative control exposure design. Fig. S3–Log minus log survival curves and p-values from Schoenfeld residuals test by maternal diabetes categories. Fig. S4–Age-specific incidence rate of epilepsy in children with 95% confidence intervals

## Data Availability

This study used individual-level data from Swedish national registers, which cannot be shared publicly due to ethical restrictions. Access to these data can be requested from Statistics Sweden and the National Board of Health and Welfare, subject to ethical approval and data use agreements. All relevant aggregate results and metadata related to this manuscript are fully reported within the article and its supplementary materials.

## Abbreviations

ADHD: Attention-deficit/hyperactivity disorder
ASD: Autism spectrum disorder
BMI: Body mass index
CI: Confidence interval
GDM: Gestational diabetes mellitus
HR: Hazard ratio
ICD: International classification of disease
LGA: Large for gestational age
SGA: Small for gestational age
PPV: Positive predictive value
T1DM: Type 1 diabetes mellitus
T2DM: Type 2 diabetes mellitus
