# Supplementary material for: Intrauterine exposure to maternal diabetes and the risk of developing epilepsy in children: a national cohort study of 2.3 million children
Source: BMC Med. 2026 Feb 14;24:123. doi: 10.1186/s12916-026-04696-0 (PMC12930888; doi:10.1186/s12916-026-04696-0)
Supplement: Supplementary file 1 — Additional file 1. Supplementary Methods Text S1-Text S2. Text S1–Supplementary methods: description of data sources. Text S2–Author-written Stata program for sequential mediation analysis in a multiple, causally ordered mediators framework [file 12916_2026_4696_MOESM1_ESM.pdf]

Additional File 1: Supplementary Methods

Text S1. Supplementary methods: description of data sources

The Swedish registers cross-linked for the analysis:

Medical Birth Register (MBR): Established in 1973, the Swedish Medical Birth Register is a national database containing comprehensive data on prenatal, obstetric, and neonatal characteristics, including maternal diagnoses. Data are routinely and prospectively collected during antenatal visits, delivery, and neonatal care, using standardized forms to ensure consistency. The MBR covers over 98% of all births in Sweden and serves as a high-quality registry for perinatal research [28].

National Patient Register (NPR): The National Patient Register comprises both inpatient and outpatient data from public and private healthcare providers. The inpatient register, initially launched in 1964 (with psychiatric diagnoses included from 1973), achieved nationwide coverage by 1987. The outpatient register, introduced in 2001, records visits to specialized outpatient care. Disease diagnoses in the NPR are categorized using the Swedish version of the International Classification of Diseases (ICD) codes. The inpatient register is highly reliable for chronic disease classification, with positive predictive values (PPVs) of approximately 85-95% for most conditions [30]. A recent evaluation by Everhov et al. (2025) reported a median PPV of 84% (interquartile range 72-93%) across inpatient and outpatient diagnoses [76].

Cause of Death Register (CDR): In operation since 1952, the Swedish Cause of Death Register provides high-quality data for research, capturing all deaths in Sweden. The CDR is linked to other national registers through unique personal identification numbers. Unlike the NPR, which uses the Swedish ICD codes, the CDR applies the international version to enable global comparison of cause-specific mortality statistics [31].

Total Population Register (TPR): Since 1968, the Total Population Register has maintained sociodemographic details, such as gender and country of birth, alongside key life events like birth, death, and migration for all residents in Sweden. The TPR enables complete follow up and censoring of individuals and thereby minimizes the risk of selection bias [32].

Education Register: Established in 1985, the Education Register is updated annually with data on individuals' educational achievements. Information is collected from over 30 sources, including educational institutions and surveys. Education is classified using the Swedish nomenclature SUN, which was later aligned with the International Standard Classification of Education (ISCED 97) in 2000 to differentiate levels and types of education [33].

Prescribed Drug Register (PDR): Established in July 2005, the Swedish Prescribed Drug Register (PDR) captures information on all outpatient prescription medications dispensed at pharmacies nationwide. The data are sourced from the Swedish eHealth Agency and are considered to be of high quality, with minimal errors and missingness. Medications in the PDR are classified using the World Health Organization’s Anatomical Therapeutic Chemical (ATC) system [34].

Multi-Generation Register (MGR): The Swedish Multi-Generation Register contains information on parents and relatives of individuals registered in Sweden since 1961 and born from 1932 onwards [35] . In the current study, the MGR was utilized to identify fathers and derive data on paternal morbidity.

Text S2. Author-written Stata program for sequential mediation analysis in a multiple, causally ordered mediators framework

```
*------------------------------------------------------------------------------------------------------------------------
*User-written Stata program for sequential mediation analysis
*Author: Muhammad Zakir Hossin
*Date: 12 October 2024
*Purpose: This user-written program has been written to sequentially estimate total, direct, and
indirect effects with multiple mediators (M1=preeclampsia, M2=preterm birth, M3=birth asphyxia),
using multiply imputed data and Cox models. The analysis assumes a causal sequence among the
mediators and the absence of exposure-mediator interactions.
*------------------------------------------------------------------------------------------------------------------------

use data_mim, clear

*Prepare the imputed data for survival analysis
mim, cat(manip) sortorder(childid): stset exitdate, enter(dob) origin(dob)
failure(epilepsy==1) scale(365.25) id(childid)

*Drop program if it already exists
capture program drop seqMed

*Define a user-written program
program seqMed, rclass

*----------------------------------------------------------------------------------------------------------------------
*Total Effect model
*----------------------------------------------------------------------------------------------------------------------
mim, storebv: stcox t1dm_m sex i.byear_cat i.edu_m i.parity  ib3.agecat_m cohabit i.country_m
smoke_m alcohol_m ib2.bmicat_m htn_m epilepsy_m  psychiat_m t1dm_f epilepsy_f psychiat_f
ib3.agecat_f, vce(cluster motherid) nolog base

matrix bb_total = e(b)
scalar b_total = bb_total[1,1]
return scalar b_total = bb_total[1,1]

*---------------------------------------------------------------------------------------------------------------------
*Direct Effect model: M1 adjusted
*---------------------------------------------------------------------------------------------------------------------
mim, storebv: stcox t1dm_m preeclamp sex i.byear_cat i.edu_m i.parity  ib3.agecat_m cohabit
i.country_m smoke_m alcohol_m ib2.bmicat_m htn_m epilepsy_m  psychiat_m t1dm_f epilepsy_f
psychiat_f ib3.agecat_f, vce(cluster motherid) nolog base

matrix bb_direct_m1 = e(b)
scalar b_NDE1 = bb_direct_m1[1,1]
return scalar b_NDE1 = bb_direct_m1[1,1]

*Indirect Effect via M1 only
scalar b_NIE1 = (b_total - b_NDE1)
return scalar b_NIE1 = (b_total - b_NDE1)

*---------------------------------------------------------------------------------------------------------------------
*Direct Effect model: M1 + M2 adjusted
*---------------------------------------------------------------------------------------------------------------------
mim, storebv: stcox t1dm_m preeclamp preterm sex i.byear_cat i.edu_m i.parity  ib3.agecat_m
cohabit i.country_m smoke_m alcohol_m ib2.bmicat_m htn_m epilepsy_m  psychiat_m t1dm_f
epilepsy_f psychiat_f ib3.agecat_f, vce(cluster motherid) nolog base

matrix bb_direct_m1m2 = e(b)
scalar b_NDE12 = bb_direct_m1m2[1,1]
return scalar b_NDE12 = bb_direct_m1m2[1,1]
```

***Indirect Effect via M1 and M2**
scalar b_NIE12 = (b_total - b_NDE12)
return scalar b_NIE12 = (b_total - b_NDE12)

*--
***Direct Effect model: M1 + M2 + M3 adjusted**
*--
mim, storebv: stcox t1dm_m preeclamp preterm asphyxia sex i.byear_cat i.edu_m i.parity ib3.agecat_m cohabit i.country_m smoke_m alcohol_m ib2.bmicat_m htn_m epilepsy_m psychiat_m t1dm_f epilepsy_f psychiat_f ib3.agecat_f, vce(cluster motherid) nolog base

matrix bb_direct123 = e(b)
scalar b_NDE123 = bb_direct123[1,1]
return scalar b_NDE123 = bb_direct123[1,1]

***Indirect Effect via M1, M2 and M3**
scalar b_NIE123 = (b_total - b_NDE123)
return scalar b_NIE123 = (b_total - b_NDE123)

*--
***Indirect Effects via M2 alone, both M2 and M3, and M3 alone**
*--

***Indirect Effect via M2 alone**
scalar b_NIE2 = (b_NDE1 - b_NDE12)
return scalar b_NIE2 = (b_NDE1 - b_NDE12)

***Indirect Effect via M2 and M3**
scalar b_NIE23 = (b_NDE1 - b_NDE123)
return scalar b_NIE23 = (b_NDE1 - b_NDE123)

***Indirect Effect via M3 alone**
scalar b_NIE3 = (b_NDE12 - b_NDE123)
return scalar b_NIE3 = (b_NDE12 - b_NDE123)

end

*--
***Bootstrap to derive confidence intervals for total, indirect, and direct effects, using 1000 replications**
*--
bootstrap r(b_total) r(b_NDE1) r(b_NIE1) r(b_NDE12) r(b_NIE12) r(b_NDE123) r(b_NIE123) r(b_NIE2) r(b_NIE23) r(b_NIE3), cluster(motherid) seed(1234587) reps(1000): seqMed

estat bootstrap, all eform

exit
