# Supplementary material for: Intrauterine exposure to maternal diabetes and the risk of developing epilepsy in children: a national cohort study of 2.3 million children
Source: BMC Med. 2026 Feb 14;24:123. doi: 10.1186/s12916-026-04696-0 (PMC12930888; doi:10.1186/s12916-026-04696-0)
Supplement: Supplementary file 2 — Additional file 2. Supplementary Tables S1-S19. Table S1–International Classification of Disease-10 codes for maternal and child morbidity and the Anatomical Therapeutic Chemicalcodes for maternal drug use. Table S2–Distribution of sample characteristics according to missing and complete data. Table S3–Odds ratios for the associations between maternal diabetes and potential mediators. Table S4– Incidence rates and hazard ratios of epilepsy in children by potential mediating variables. Table S5–Incidence rates and subdistribution hazard ratios of epilepsy in children by maternal and paternal diabetes status, estimated through competing risk analysis including both live births and stillbirths. Table S6–E-values for the total, direct, and indirect effects of maternal T1DM and T2DM and for the mediator-outcome estimates. Table S7–Sequential mediation of the associations of maternal T1DM and T2DM with child epilepsy via preeclampsia, gestational age, and birth asphyxia. Table S8–Hazard ratios of epilepsy in children by maternal and paternal diabetes status, using the spline terms to fit continuous confounders. Table S9–Sex-stratified incidence rates and hazard ratios of epilepsy in children by maternal and paternal diabetes status. Table S10– Incidence rates and hazard ratios of epilepsy in children by maternal and paternal diabetes status, after excluding children with diabetes or parental epilepsy. Table S11–Incidence rates and hazard ratios of epilepsy in children by maternal diabetes status, reclassified based on insulin use. Table S12–Incidence rates and hazard ratios of epilepsy in children by maternal diabetes status, after excluding overlapping diagnoses of maternal T1DM and T2DM. Table S13–Incidence rates and hazard ratios of epilepsy in children by parental diabetes status, restricting maternal T1DM and T2DM diagnoses to the preconception period. Table S14–Incidence rates and hazard ratios of epilepsy in children by maternal use of diabetes medications during p [file 12916_2026_4696_MOESM2_ESM.pdf]

Additional File 2: Supplementary Tables

Table S1. International Classification of Disease (ICD)-10 codes for maternal and child morbidity and the Anatomical Therapeutic Chemical (ATC) codes for maternal drug use

Maternal diseases	ICD-10/ATC code	Data source
T1DM	E10, O240	MBR + NPR
T2DM	E11, O241	MBR + NPR
GDM	O244	MBR + NPR
Preeclampsia	O14, O15	MBR
Chronic hypertension	I10-I15, O10 and O11	MBR + checkbox[#]
Psychiatric disorders	F00-F99	NPR
Epilepsy	G40, G41	NPR
Alcohol use disorder in pregnancy	F10, O993, O354, Q860	NPR
**Maternal use of diabetes drugs***		
Insulin	A10A, A10AB01, A10AB04, A10AB05, A10AB06, A10AC01, A10AD01, A10AD04, A10AD05, A10AE04, A10AE05, A10AE06, A10AE56, A10AF01	PDR
Oral antidiabetics	A10BA02, A10BB01, A10BB07, A10BB12, A10BD03, A10BD05, A10BD07, A10BD20, A10BF01, A10BG02, A10BG03, A10BH01, A10BH02, A10BH03, A10BH05, A10BJ01, A10BJ02, A10BJ03, A10BJ05, A10BJ06, A10BK01, A10BK02, A10BK03, A10BX02, A10BX03.	PDR
**Child diseases**		
Epilepsy	G40, G41	NPR
Focal epilepsy	G400-G402	NPR
Generalized epilepsy	G403	NPR
Birth asphyxia		
Meconium aspiration	P24	MBR + NPR
Convulsion/seizure	P90	MBR + NPR
Hypoxic ischemic encephalopathy	P913-P916	MBR + NPR
Major malformations	Q7550, Q175, Q180, Q181, Q249, Q250, Q270, Q289, Q314, Q315, Q320, Q381, Q523, Q53, Q65, Q665, Q666, Q667, Q668, Q669, Q690, Q699, Q703, Q760, Q799, Q825, Q829	MBR + NPR

Note: GDM, gestational diabetes mellitus; ICD, the International Classification of Disease; MBR, Medical Birth Register; NPR, National Patient Register; PDR, Prescription Drug Register; T1DM, type 1 diabetes mellitus; T2DM, type 1 diabetes mellitus.

*Information on diabetes drugs was derived for sensitivity analyses from the Prescription Drug Register available since July 2005, using the Anatomical Therapeutic Chemical (ATC) codes.

[#]Chronic hypertension is also recorded in a checkbox in the prenatal record at first prenatal visit

Table S2. Distribution of sample characteristics according to missing and complete data

Sample characteristics	**Total** (N=2,305,051)	**Missing data** (N=260,444)[1]	**Complete data** (N=2,044,607)[2]	**P-value**[3]
**Infant characteristics** % (N)				
Sex				0.40
Male	51.1 (1,177,861)	51.2 (133,285)	51.1 (1,044,576)	
Female	48.9 (1,127,190)	48.8 (127,159)	48.9 (1,000,031)	
Birth year				<0.001
1998-2002	17.9 (412,722)	25.6 (66,596)	16.9 (346,126)	
2003-2007	20.2 (464,794)	25.0 (65,021)	19.6 (399,773)	
2008-2012	21.8 (503,103)	14.5 (37,713)	22.8 (465,390)	
2013-2017	22.4 (516,445)	17.4 (45,336)	23.0 (471,109)	
2018-2021	17.7 (407,987)	17.6 (45,778)	17.7 (362,209)	
Preterm birth				<0.001
No	95.6 (2,202,806)	94.2 (245,311)	95.7 (1,957,495)	
Yes	4.4 (102,245)	5.8 (15,133)	4.3 (87,112)	
Birthweight for gestational age				<0.001
SGA	6.2 (142,200)	6.8 (17,586)	6.1 (124,614)	
AGA	81.7 (1,879,781)	81.1 (210,455)	81.8 (1,669,326)	
LGA	12.1 (277,919)	12.1 (31,503)	12.1 (246,416)	
Birth asphyxia				<0.001
No	99.5 (2,293,517)	99.4 (258,856)	99.5 (2,034,661)	
Yes	0.5 (11,534)	0.6 (1,588)	0.5 (9,946)	
**Maternal characteristics %** (N)				
Age at childbirth (years)				<0.001
≤19	1.3 (30,403)	2.0 (5,164)	1.2 (25,239)	
20-24	12.1 (277,968)	12.7 (33,050)	12.0 (244,918)	
25-29	31.1 (716,257)	29.2 (76,076)	31.3 (640,181)	
30-34	35.0 (805,797)	34.5 (89,834)	35.0 (715,963)	
≥35	20.6 (474,626)	21.6 (56,320)	20.5 (418,306)	
Parity				<0.001
1	43.4 (1,000,628)	43.7 (113,706)	43.4 (886,922)	
2	37.3 (860,726)	35.8 (93,206)	37.5 (767,520)	
3	13.5 (310,749)	13.5 (35,175)	13.5 (275,574)	
≥4	5.8 (132,948)	7.0 (18,357)	5.6 (114,591)	
Country of birth				<0.001
Sweden	76.8 (1,768,112)	69.7 (180,133)	77.7 (1,587,979)	
Other Nordic country	1.5 (35,348)	2.4 (6,170)	1.4 (29,178)	
Non-Nordic country	21.7 (499,608)	27.9 (72,158)	20.9 (427,450)	
Level of education (years)				<0.001
≤9	8.4 (190,343)	8.7 (19,698)	8.3 (170,645)	
10-11	11.2 (254,716)	12.7 (28,902)	11.0 (225,814)	
12	26.1 (594,172)	24.2 (55,063)	26.4 (539,109)	
13-14	14.7 (334,141)	15.0 (34,123)	14.7 (300,018)	
≥15	39.6 (898,838)	39.5 (89,817)	39.6 (809,021)	
Cohabitation with partner				<0.001
No	5.2 (114,985)	7.0 (10,413)	5.1 (104,572)	

Yes	94.8 (2,078,116)	93.0 (138,081)	94.9 (1,940,035)	
Smoking in early pregnancy				<0.001
No	92.5 (2,053,286)	91.4 (159,838)	92.6 (1,893,448)	
Yes	7.5 (166,289)	8.6 (15,130)	7.4 (151,159)	
BMI in early pregnancy (kg/m2)				<0.001
Underweight (≤18.4)	2.4 (51,828)	3.2 (2,469)	2.4 (49,359)	
Normal weight (18.5-24.9)	59.5 (1,261,814)	56.0 (43,109)	59.6 (1,218,705)	
Overweight (25-29.9)	25.5 (541,482)	26.4 (20,347)	25.5 (521,135)	
Obese (≥30)	12.6 (266,415)	14.3 (11,007)	12.5 (255,408)	
Alcohol use disorder in pregnancy				
No	98.6 (2,272,439)	98.8 (257,313)	98.6 (2,015,126)	<0.001
Yes	1.4 (32,612)	1.2 (3,131)	1.4 (29,481)	
Epilepsy				<0.001
No	99.4 (2,291,840)	99.5 (259,139)	99.4 (2,032,701)	
Yes	0.6 (13,211)	0.5 (1,305)	0.6 (11,906)	
Psychiatric disorders				<0.001
No	90.9 (2,094,341)	92.3 (240,306)	90.7 (1,854,035)	
Yes	9.1 (210,710)	7.7 (20,138)	9.3 (190,572)	
Pregestational hypertension				<0.001
No	99.3 (2,289,156)	99.4 (258,853)	99.3 (2,030,303)	
Yes	0.7 (15,895)	0.6 (1,591)	0.7 (14,304)	
Preeclampsia				0.92
No	97.3 (2,241,775)	97.3 (253,302)	97.3 (1,988,473)	
Yes	2.7 (63,276)	2.7 (7,142)	2.7 (56,134)	

Note: AGA, Appropriate for gestational age; BMI, body mass index; LGA, Large for gestational age; SGA, Small for gestational age.

[1]The sample with data missing on any of the study variables. [2]The sample with complete data on all study variables. [3]Chi-squared p-value testing for differences in the proportions of missing data across groups.

Table S3. Odds ratios for the associations between maternal diabetes and potential mediators

Potential mediators	Model	Maternal diabetes		
		T1DM	T2DM	GDM
		OR (95% CI)	OR (95% CI)	OR (95% CI)
Preeclampsia				
	Model 1	5.73 (5.46, 6.02)	2.82 (2.49, 3.19)	2.01 (1.92, 2.11)
	Model 2	5.26 (4.98, 5.55)	2.87 (2.51, 3.28)	1.79 (1.70, 1.88)
Preterm birth				
	Model 1	5.63 (5.40, 5.86)	2.99 (2.71, 3.30)	1.70 (1.64, 1.77)
	Model 2	5.25 (5.02, 5.49)	2.58 (2.32, 2.88)	1.67 (1.60, 1.75)
Birth-asphyxia*				
	Model 1	2.97 (2.58, 3.41)	2.85 (2.17, 3.73)	1.34 (1.18, 1.53)
	Model 2	2.70 (2.33, 3.13)	2.30 (1.71, 3.08)	1.15 (1.00, 1.32)
SGA				
	Model 1	0.44 (0.40, 0.48)	0.93 (0.81, 1.07)	0.84 (0.80, 0.88)
	Model 2	0.39 (0.34, 0.43)	0.80 (0.69, 0.93)	0.77 (0.73, 0.81)
LGA				
	Model 1	8.51 (8.23, 8.80)	3.45 (3.22, 3.70)	2.52 (2.46, 2.58)
	Model 2	8.52 (8.21, 8.84)	3.07 (2.85, 3.31)	2.33 (2.27, 2.39)

Note: CI, confidence interval; GDM, Gestational diabetes mellitus; LGA, Large for gestational age; OR, hazard ratio; SGA, Small for gestational age; T1DM, type 1 diabetes mellitus; T2DM, type 2 diabetes mellitus.
*Birth-asphyxia was defined as any of the following complications: low Apgar score, convulsions, meconium aspiration, and hypoxic ischemic encephalopathy.
Model 1 shows the unadjusted ORs. **Model 2** was adjusted for infant sex and birth year; maternal age at childbirth, parity, country of birth, educational level, cohabitation status, smoking, body mass index, alcohol use disorders, chronic hypertension, history of epilepsy, and psychiatric disorders; and paternal age at childbirth, T1DM and T2DM, history of epilepsy, and psychiatric disorders.

Table S4. Incidence rates and hazard ratios of epilepsy in children by potential mediating variables

Potential mediators	Outcome: Epilepsy in children No. of events	Rates[1]	Model 1 HR (95% CI)	Model 2 HR (95% CI)
Maternal Preeclampsia				
No	18,294	63.1	1.00 (Ref.)	1.00 (Ref.)
Yes	674	82.0	1.30 (1.21, 1.40)	1.23 (1.14, 1.33)
Preterm birth				
No	17,587	61.8	1.00 (Ref.)	1.00 (Ref.)
Yes	1381	102.9	1.67 (1.58, 1.76)	1.58 (1.49, 1.67)
Birth-asphyxia*				
No	18,052	60.8	1.00 (Ref.)	1.00 (Ref.)
Yes	916	719.7	11.85 (11.09, 12.66)	10.98 (10.26, 11.74)
Birthweight for gestational age				
SGA	1,484	82.4	1.35 (1.28, 1.42)	1.31 (1.24, 1.38)
AGA	14,795	61.1	1.00 (Ref.)	1.00 (Ref.)
LGA	2,339	63.3	1.03 (0.99, 1.08)	0.99 (0.95, 1.04)

Note: AGA, Appropriate for gestational age; CI, confidence interval; HR, hazard ratio; LGA, Large for gestational age; SGA, Small for gestational age; T1DM, type 1 diabetes mellitus; T2DM, type 2 diabetes mellitus.

[1]Crude incidence rates per 100,000 child-years.

*Birth-asphyxia was defined as any of the following complications: low Apgar score, convulsions, meconium aspiration, and hypoxic ischemic encephalopathy.

The HRs were pooled from 12 multiply imputed datasets. **Model 1** shows the unadjusted HRs. **Model 2** was adjusted for infant sex and birth year; maternal T1DM, T2DM, preeclampsia, age at childbirth, parity, country of birth, educational level, cohabitation status, smoking, body mass index, alcohol use disorders, chronic hypertension, history of epilepsy, and psychiatric disorders; and paternal age at childbirth, T1DM and T2DM, history of epilepsy, and psychiatric disorders.

Table S5. Incidence rates and subdistribution hazard ratios of epilepsy in children by maternal and paternal diabetes status, estimated through competing risk analysis including both live births and stillbirths#

	**Outcome: Epilepsy in children**			
**Exposure**	No. of epilepsy	Rates*	Model 1 HR (95% CI)	Model 2 HR (95% CI)
**Maternal diabetes**				
Unexposed	18,752	61.7	1.00 (Ref.)	1.00 (Ref.)
T1DM	156	86.6	1.39 (1.27, 1.68)	1.31 (1.10, 1.53)
T2DM	41	106.7	1.47 (1.21, 2.14)	1.42 (1.02, 1.97)
GDM	260	72.9	1.16 (1.04, 1.30)	1.11 (0.97, 1.26)
**Paternal diabetes**				
Unexposed	19,071	62.0	1.00 (Ref.)	1.00 (Ref.)
T1DM	100	65.0	1.03 (0.84, 1.24)	1.00 (0.81, 1.23)
T2DM	38	66.6	1.04 (0.76, 1.43)	0.92 (0.65, 1.30)

Note: CI, confidence interval; GDM, Gestational diabetes mellitus; HR, hazard ratio; T1DM, type 1 diabetes mellitus; T2DM, type 2 diabetes mellitus.

#This analysis was based on both stillbirths and live-births, with follow up started from the 22nd week of gestation rather than from birth (N=2,338,627). Maternal-child data linkage was performed using pregnancy ID, instead of mother ID.

*Crude incidence rates per 100,000 person-years.

The HRs were pooled from 12 multiply imputed datasets. **Model 1** shows the unadjusted HRs. **Model 2** was adjusted for infant sex and birth year; maternal age at childbirth, parity, country of birth, educational level, cohabitation status, smoking, body mass index, alcohol use disorders, chronic hypertension, history of epilepsy, and psychiatric disorders; and paternal age at childbirth, history of epilepsy, and psychiatric disorders. Maternal and paternal diabetes were mutually adjusted for each other.

Table S6. E-values for the total, direct, and indirect effects of maternal T1DM and T2DM and for the mediator-outcome estimates

Pathway	Association	Estimate (HR, 95% CI)	E-value
T1DM: Exposure-outcome pathway			
	Total Effect	1.30 (1.09, 1.51)	1.92
	Direct Effect	1.12 (0.95, 1.32)	1.49
	Indirect Effect (via preeclampsia, preterm birth, and birth asphyxia)	1.17 (1.13, 1.20)	1.62
	Indirect Effect (via preterm birth and birth asphyxia)	1.14 (1.11, 1.17)	1.54
T2DM: Exposure-outcome pathway			
	Total Effect	1.40 (1.01, 1.96)	2.15
	Direct Effect	1.21 (0.86, 1.68)	1.71
	Indirect Effect (via preeclampsia, preterm birth, and birth asphyxia)	1.16 (1.09, 1.24)	1.59
	Indirect Effect (via preterm birth and birth asphyxia)	1.11 (1.04, 1.18)	1.46
Mediator-outcome pathway			
	Preeclampsia → Epilepsy	1.23 (1.14, 1.33)	1.76
	Preterm birth → Epilepsy	1.58 (1.49, 1.67)	2.54
	Birth asphyxia → Epilepsy	10.98 (10.27, 11.74)	21.45

Note: The E-values were calculated using the formula: HR + square root {HR * (HR-1)}

Table S7. Sequential mediation of the associations of maternal T1DM and T2DM with child epilepsy via preeclampsia, gestational age, and birth asphyxia

	**(i) Mediation by preeclampsia**	
**Mediation parameters**	**T1DM** HR (95% CI)	**T2DM** HR (95% CI)
Total Effect	1.30 (1.10, 1.52)	1.40 (1.02, 1.97)
Direct Effect	1.26 (1.07, 1.49)	1.33 (0.95, 1.86)
Indirect Effect	1.03 (1.02, 1.04)	1.05 (1.04, 1.07)
Proportion Mediated (%)	10.6	14.7
	**(ii) Joint mediation by preeclampsia and gestational age**	
	**T1DM** HR (95% CI)	**T2DM** HR (95% CI)
Total Effect	1.30 (1.10, 1.52)	1.40 (1.02, 1.97)
Direct Effect	1.16 (0.99, 1.36)	1.26 (0.90, 1.76)
Indirect Effect	1.12 (1.10, 1.14)	1.11 (1.09, 1.13)
Proportion Mediated (%)	44.1	33.9
	**(iii) Joint mediation by preeclampsia, gestational age, and birth asphyxia**	
	**T1DM** HR (95% CI)	**T2DM** HR (95% CI)
Total Effect	1.30 (1.10, 1.52)	1.40 (1.01, 1.96)
Direct Effect	1.09 (0.91, 1.27)	1.19 (0.85, 1.67)
Indirect Effect	1.19 (1.15, 1.22)	1.18 (1.11, 1.25)
Proportion Mediated (%)	68.2	48.6
	**(iv) Mediation by gestational age alone**	
	**T1DM** HR (95% CI)	**T2DM** HR (95% CI)
Indirect Effect	1.09 (1.08, 1.10)	1.06 (1.04, 1.07)
Proportion Mediated (%)	33.5	16.0
	**(v) Joint mediation by gestational age and birth asphyxia**	
	**T1DM** HR (95% CI)	**T2DM** HR (95% CI)
Indirect Effect	1.16 (1.12, 1.19)	1.12 (1.06, 1.18)
Proportion Mediated (%)	57.6	33.9
	**(vi) Mediation by birth asphyxia alone**	
	**T1DM** HR (95% CI)	**T2DM** HR (95% CI)
Indirect Effect	1.06 (1.04, 1.08)	1.06 (1.00, 1.12)
Proportion Mediated (%)	24.1	17.9

Note: CI, confidence interval; HR, hazard ratio; T1DM, type 1 diabetes mellitus; T2DM, type 2 diabetes mellitus.
Restricted cubic splines (5 knots) were used for gestational age to account for nonlinearity.
The 95% CIs are percentile based, obtained by bootstrapping with 1000 replications.
The Proportion Mediated is a ratio of the Indirect and Total Effects (i.e., Indirect Effect/Total Effect), calculated on the log HR scale and expressed in percentage.
T1DM and T2DM were used as binary exposures (No/Yes).
The HRs were adjusted for infant sex and birth year; maternal age at childbirth, parity, country of birth, educational level, cohabitation status, smoking, body mass index, alcohol use disorder, chronic hypertension, history of epilepsy, and psychiatric disorders; and paternal age at childbirth, paternal T1DM or T2DM, history of epilepsy, and psychiatric disorders.

Table S8. Hazard ratios of epilepsy in children by maternal and paternal diabetes status, using the spline terms to fit continuous confounders

Exposure	Outcome: Epilepsy in children			
	No. of epilepsy	Rates*	Model 1<br>HR (95% CI)	Model 2<br>HR (95% CI)
**Maternal diabetes**				
Unexposed	18,518	63.3	1.00 (Ref.)	1.00 (Ref.)
T1DM	156	89.8	1.41 (1.20, 1.64)	1.30 (1.10, 1.54)
T2DM	37	102.9	1.56 (1.13, 2.15)	1.38 (0.99, 1.93)
GDM	257	76.2	1.17 (1.04, 1.32)	1.09 (0.96, 1.25)
**Paternal diabetes**				
Unexposed	18,830	63.6	1.00 (Ref.)	1.00 (Ref.)
T1DM	100	67.2	1.03 (0.85, 1.25)	0.98 (0.80, 1.21)
T2DM	38	68.9	1.04 (0.76, 1.43)	0.93 (0.66, 1.30)

Note: CI, confidence interval; GDM, Gestational diabetes mellitus; HR, hazard ratio; T1DM, type 1 diabetes mellitus; T2DM, type 2 diabetes mellitus.

*Crude incidence rates per 100,000 person-years.

The HRs were pooled from 12 multiply imputed datasets. **Model 1** shows the unadjusted HRs. **Model 2** was adjusted for infant sex and birth year; maternal age at childbirth, parity, country of birth, years of education, cohabitation status, smoking, body mass index, alcohol use disorders, chronic hypertension, history of epilepsy, and psychiatric disorders; and paternal age at childbirth, history of epilepsy, and psychiatric disorders. Maternal and paternal diabetes were mutually adjusted for each other. Restricted cubic splines were used for birth year, maternal age, parity, years of education and BMI to account for possible non-linearity.

Table S9. Sex-stratified incidence rates and hazard ratios of epilepsy in children by maternal and paternal diabetes status

**Females** (N=1,127,190)				
	**Outcome: Epilepsy in children**			
**Exposure**	No. of epilepsy	Rates*	Model 1 HR (95% CI)	Model 2 HR (95% CI)
**Maternal diabetes**				
Unexposed	8,649	60.5	1.00 (Ref.)	1.00 (Ref.)
T1DM	71	83.5	1.37 (1.08, 1.73)	1.25 (0.99, 1.57)
T2DM	16	88.9	1.41 (0.87, 2.30)	1.23 (0.75, 2.00)
GDM	109	66.1	1.07 (0.88, 1.29)	1.00 (0.82, 1.20)
**Paternal diabetes**				
Unexposed	8,777	60.7	1.00 (Ref.)	1.00 (Ref.)
T1DM	46	63.2	1.02 (0.76, 1.36)	0.98 (0.74, 1.31)
T2DM	22	81.1	1.29 (0.85, 1.96)	1.18 (0.78, 1.80)
**Males** (N=1,177,861)				
**Maternal diabetes**				
Unexposed	9,868	66.0	1.00 (Ref.)	1.00 (Ref.)
T1DM	85	95.9	1.44 (1.16, 1.78)	1.33 (1.08, 1.65)
T2DM	21	116.9	1.69 (1.10, 2.60)	1.41 (0.91, 2.16)
GDM	148	85.8	1.26 (1.07, 1.48)	1.11 (0.95, 1.32)
**Paternal diabetes**				
Unexposed	10,052	66.4	1.00 (Ref.)	1.00 (Ref.)
T1DM	54	70.9	1.04 (0.80, 1.36)	1.02 (0.78, 1.33)
T2DM	16	57.1	0.82 (0.50, 1.35)	0.71 (0.43, 1.15)

Note: CI, confidence interval; GDM, Gestational diabetes mellitus; HR, hazard ratio; T1DM, type 1 diabetes mellitus; T2DM, type 2 diabetes mellitus.

*Crude incidence rates per 100,000 person-years.

P-value for maternal T1DM*infant sex: 0.698, P-value for maternal T2DM*infant sex: 0.683, P-value for GDM*infant sex: 0.222, P-value for overall maternal diabetes*infant sex: 0.617

The HRs were pooled from 12 multiply imputed datasets. **Model 1** shows the unadjusted HRs. **Model 2** was adjusted for birth year and maternal age at childbirth, parity, country of birth, educational level, cohabitation status, smoking, body mass index, alcohol use disorders, chronic hypertension, history of epilepsy, and psychiatric disorders; and paternal age at childbirth, history of epilepsy, and psychiatric disorders. Maternal and paternal diabetes were mutually adjusted for each other.

Table S10. Incidence rates and hazard ratios of epilepsy in children by maternal and paternal diabetes status, after excluding children with diabetes or parental epilepsy

	**Outcome: Epilepsy in children**			
**Exposure**	No. of epilepsy	Rates*	Model 1 HR (95% CI)	Model 2 HR (95% CI)
**Maternal diabetes**				
Unexposed	17,684	61.5	1.00 (Ref.)	1.00 (Ref.)
T1DM	138	84.6	1.36 (1.15, 1.61)	1.28 (1.08, 1.51)
T2DM	35	100.8	1.62 (1.16, 2.26)	1.41 (1.01, 1.96)
GDM	242	73.3	1.16 (1.02, 1.32)	1.07 (0.94, 1.21)
**Paternal diabetes**				
Unexposed	17,974	61.8	1.00 (Ref.)	1.00 (Ref.)
T1DM	90	65.0	1.03 (0.83, 1.26)	1.01 (0.82, 1.25)
T2DM	35	65.6	1.02 (0.73, 1.42)	0.93 (0.67, 1.29)

Note: CI, confidence interval; GDM, Gestational diabetes mellitus; HR, hazard ratio; T1DM, type 1 diabetes mellitus; T2DM, type 2 diabetes mellitus.

*Crude incidence rates per 100,000 person-years.

The HRs were pooled from 12 multiply imputed datasets. **Model 1** shows the unadjusted HRs. **Model 2** was adjusted for infant sex and birth year; maternal age at childbirth, parity, country of birth, educational level, cohabitation status, smoking, body mass index, alcohol use disorders, chronic hypertension, and history of psychiatric disorders; and paternal age at childbirth and history psychiatric disorders. Maternal and paternal diabetes were mutually adjusted for each other.

Table S11. Incidence rates and hazard ratios of epilepsy in children by maternal diabetes status, reclassified based on insulin use

Exposure	Outcome: Epilepsy in children			
	No. of epilepsy	Rates*	Model 1 HR (95% CI)	Model 2 HR (95% CI)
**Maternal diabetes**				
Unexposed	18,517	63.3	1.00 (Ref.)	1.00 (Ref.)
T1DM	153	90.3	1.42 (1.21, 1.66)	1.31 (1.11, 1.56)
T2DM	40	99.3	1.50 (1.10, 2.05)	1.37 (0.99, 1.88)
GDM	257	76.2	1.17 (1.03, 1.32)	1.10 (0.95, 1.25)

Note: CI, confidence interval; GDM, Gestational diabetes mellitus; HR, hazard ratio; T1DM, type 1 diabetes mellitus; T2DM, type 2 diabetes mellitus.

*Crude incidence rates per 100,000 person-years.

The HRs were pooled from 12 multiply imputed datasets. **Model 1** shows the unadjusted HRs. **Model 2** was adjusted for infant sex and birth year; maternal age at childbirth, parity, country of birth, educational level, cohabitation status, smoking, body mass index, alcohol use disorders, chronic hypertension, history of epilepsy, and psychiatric disorders; and paternal age at childbirth, T1DM and T2DM, history of epilepsy, and psychiatric disorders.

Table S12. Incidence rates and hazard ratios of epilepsy in children by maternal diabetes status, after excluding overlapping diagnoses of maternal T1DM and T2DM

	**Outcome: Epilepsy in children**			
**Exposure**	No. of epilepsy	Rates*	Model 1 HR (95% CI)	Model 2 HR (95% CI)
**Maternal diabetes**				
Unexposed	18,517	63.3	1.00 (Ref.)	1.00 (Ref.)
T1DM	135	95.3	1.50 (1.27, 1.78)	1.40 (1.18, 1.66)
T2DM	30	116.0	1.75 (1.22, 2.50)	1.49 (1.04, 2.13)
GDM	257	76.2	1.17 (1.03, 1.32)	1.08 (0.95, 1.22)

Note: CI, confidence interval; GDM, Gestational diabetes mellitus; HR, hazard ratio; T1DM, type 1 diabetes mellitus; T2DM, type 2 diabetes mellitus.
*Crude incidence rates per 100,000 person-years.
The HRs were pooled from 12 multiply imputed datasets. **Model 1** shows the unadjusted HRs. **Model 2** was adjusted for infant sex and birth year; maternal age at childbirth, parity, country of birth, educational level, cohabitation status, smoking, body mass index, alcohol use disorders, chronic hypertension, history of epilepsy, and psychiatric disorders; and paternal age at childbirth, T1DM and T2DM, history of epilepsy, and psychiatric disorders.

Table S13. Incidence rates and hazard ratios of epilepsy in children by parental diabetes status, restricting maternal T1DM and T2DM diagnoses to the preconception period

Exposure	Outcome: Epilepsy in children			
	No. of epilepsy	Rates*	Model 1 HR (95% CI)	Model 2 HR (95% CI)
**Maternal diabetes**				
Unexposed	18,541	63.3	1.00 (Ref.)	1.00 (Ref.)
T1DM	138	91.9	1.44 (1.22, 1.70)	1.32 (1.10, 1.59)
T2DM	31	94.7	1.42 (1.01, 2.02)	1.29 (0.89, 1.85)
GDM	257	76.2	1.17 (1.04, 1.32)	1.09 (0.96, 1.25)
**Paternal diabetes**				
Unexposed	18,830	63.6	1.00 (Ref.)	1.00 (Ref.)
T1DM	100	67.2	1.03 (0.85, 1.25)	0.98 (0.80, 1.21)
T2DM	38	68.9	1.04 (0.76, 1.43)	0.93 (0.66, 1.30)

Note: CI, confidence interval; GDM, Gestational diabetes mellitus; HR, hazard ratio; T1DM, type 1 diabetes mellitus; T2DM, type 2 diabetes mellitus.
*Crude incidence rates per 100,000 person-years.
The HRs were pooled from 12 multiply imputed datasets. **Model 1** shows the unadjusted HRs. **Model 2** was adjusted for infant sex and birth year; maternal age at childbirth, parity, country of birth, years of education, cohabitation status, smoking, body mass index, alcohol use disorders, chronic hypertension, history of epilepsy, and psychiatric disorders; and paternal age at childbirth, history of epilepsy, and psychiatric disorders. Maternal and paternal diabetes were mutually adjusted for each other.

Table S14. Incidence rates and hazard ratios of epilepsy in children by maternal use of diabetes medications during pregnancy[#]

**Diabetes medications**	**Outcome: Epilepsy in children**			
	No. of events	Rates[*]	Model 1 HR (95% CI)	Model 2 HR (95% CI)
Insulin use in pregnancy				
No	165	78.2	1.00 (Ref.)	1.00 (Ref.)
Yes	129	87.8	1.12 (0.89, 1.42)	1.12 (0.83, 1.51)
Oral antidiabetics use in pregnancy				
No	265	80.3	1.00 (Ref.)	1.00 (Ref.)
Yes	29	104.6	1.25 (0.85, 1.85)	1.13 (0.72, 1.75)

Note: CI, confidence interval; HR, hazard ratio.

[#]This analysis included only children born to mothers with diabetes from 2005 onward, corresponding to the period when prescription drug data became available in Sweden (N=45,565). The purpose of restricting the analysis to children of mothers with diabetes was to account for confounding by indication.

[*]Crude incidence rates per 100,000 child-years.

Model 1 shows the unadjusted HRs. **Model 2** was adjusted for infant sex and birth year; types of maternal diabetes, preeclampsia, age at childbirth, parity, country of birth, educational level, cohabitation status, smoking, body mass index, alcohol use disorders, chronic hypertension, history of epilepsy, and psychiatric disorders; and paternal age at childbirth, T1DM and T2DM, history of epilepsy, and psychiatric disorders.

Table S15. Incidence rates and hazard ratios of epilepsy in children by duration of pre-gestational type 1 and type 2 diabetes

Exposure	Outcome: Epilepsy in children			
	No. of events	Rates*	Model 1 HR (95% CI)	Model 2 HR (95% CI)
**Diagnosis of maternal T1DM**				
No T1DM	18,809	63.5	1.00 (Ref.)	1.00 (Ref.)
T1DM <5 years before childbirth	88	83.5	1.34 (1.08, 1.65)	1.23 (1.00, 1.52)
T1DM 5–10 years before childbirth	37	95.5	1.47 (1.06, 2.02)	1.35 (0.98, 1.87)
T1DM >10 years before childbirth	34	101.0	1.46 (1.04, 2.04)	1.31 (0.93, 1.83)
**Diagnosis of maternal T2DM**				
No T2DM	18,911	63.6	1.00 (Ref.)	1.00 (Ref.)
T2DM <5 years before childbirth	50	95.5	1.45 (1.10, 1.92)	1.35 (1.01, 1.80)
T2DM 5–10 years before childbirth	<10!	-	-	-
T2DM >10 years before childbirth	<10!	-	-	-

Note: CI, confidence interval; HR, hazard ratio; T1DM, type 1 diabetes mellitus; T2DM, type 2 diabetes mellitus.
*Crude incidence rates per 100,000 person-years.
! Exact count is suppressed for confidentiality reason. -Not estimated due to insufficient number of events.
The HRs were pooled from 12 multiply imputed datasets. **Model 1** shows the unadjusted HRs. **Model 2** was adjusted for infant sex and birth year; maternal age at childbirth, parity, country of birth, educational level, cohabitation status, smoking, body mass index, alcohol use disorders, chronic hypertension, history of epilepsy, and psychiatric disorders; and paternal age at childbirth, T1DM and T2DM, history of epilepsy, and psychiatric disorders.

Table S16. Incidence rates and hazard ratios of epilepsy by maternal diabetes status among children of Swedish-born mothers

Exposure	Outcome: Epilepsy in children			
	No. of epilepsy	Rates*	Model 1 HR (95% CI)	Model 2 HR (95% CI)
**Maternal diabetes**				
Unexposed	14,905	63.6	1.00 (Ref.)	1.00 (Ref.)
T1DM	138	92.8	1.44 (1.22, 1.71)	1.33 (1.13, 1.58)
T2DM	22	103.7	1.72 (1.14, 2.62)	1.40 (0.92, 2.13)
GDM	141	70.7	1.09 (0.92, 1.28)	0.97 (0.82, 1.15)

Note: CI, confidence interval; GDM, Gestational diabetes mellitus; HR, hazard ratio; T1DM, type 1 diabetes mellitus; T2DM, type 2 diabetes mellitus.
*Crude incidence rates per 100,000 person-years.
The HRs were pooled from 12 multiply imputed datasets. **Model 1** shows the unadjusted HRs. **Model 2** was adjusted for infant sex and birth year; maternal age at childbirth, parity, educational level, cohabitation status, smoking, body mass index, alcohol use disorders, chronic hypertension, history of epilepsy, and psychiatric disorders; and paternal at childbirth, T1DM and T2DM, history of epilepsy, and psychiatric disorders.

Table S17. Incidence rates and hazard ratios of epilepsy by maternal diabetes status before the revised GDM criteria in 2018

	Outcome: Epilepsy in children			
**Exposure**	No. of epilepsy	Rates*	Model 1 HR (95% CI)	Model 2 HR (95% CI)
**Maternal diabetes**				
Unexposed	17,380	62.6	1.00 (Ref.)	1.00 (Ref.)
T1DM	147	90.4	1.43 (1.22, 1.69)	1.33 (1.13, 1.56)
T2DM	31	98.9	1.53 (1.07, 2.17)	1.31 (0.92, 1.87)
GDM	204	72.8	1.15 (1.00, 1.32)	1.07 (0.93, 1.22)

Note: CI, confidence interval; GDM, Gestational diabetes mellitus; HR, hazard ratio; T1DM, type 1 diabetes mellitus; T2DM, type 2 diabetes mellitus.

*Crude incidence rates per 100,000 person-years.

The HRs were pooled from 12 multiply imputed datasets. **Model 1** shows the unadjusted HRs. **Model 2** was adjusted for infant sex and birth year; mother's age at childbirth, parity, country of birth, educational level, cohabitation status, smoking, body mass index, alcohol use disorders, chronic hypertension, history of epilepsy, and psychiatric disorders; and paternal age at childbirth, T1DM and T2DM, history of epilepsy, and psychiatric disorders.

Table S18. Incidence rates and hazard ratios of epilepsy by severity of maternal GDM[#]

Exposure	Outcome: Epilepsy in children No. of epilepsy	Rates*	Model 1 HR (95% CI)	Model 2 HR (95% CI)
**GDM severity**				
No GDM	11,238	66.8	1.00 (Ref.)	1.00 (Ref.)
GDM without medication	137	79.2	1.16 (0.98, 1.37)	1.08 (0.90, 1.29)
GDM with medication	46	96.8	1.40 (1.04, 1.86)	1.11 (0.81, 1.54)

Note: CI, confidence interval; GDM, Gestational diabetes mellitus; HR, hazard ratio.

[#]This analysis was restricted to children born after 2004, coinciding with the availability of prescription drugs information in Sweden (N=1,710,867).

*Crude incidence rates per 100,000 person-years.

The HRs were pooled from 12 multiply imputed datasets. **Model 1** shows the unadjusted HRs. **Model 2** was adjusted for infant sex and birth year; mother's age at childbirth, parity, country of birth, educational level, cohabitation status, smoking, body mass index, alcohol use disorders, chronic hypertension, T1DM and T2DM, history of epilepsy, and psychiatric disorders; and paternal age at childbirth, T1DM and T2DM, history of epilepsy, and psychiatric disorders.

Table S19. Incidence rates and hazard ratios of epilepsy in children by maternal and paternal diabetes status, with major malformations included (N=2,403,421)

Exposure	Outcome: Epilepsy in children			
	No. of epilepsy	Rates*	Model 1 HR (95% CI)	Model 2 HR (95% CI)
**Maternal diabetes**				
Unexposed	21,000	69.3	1.00 (Ref.)	1.00 (Ref.)
T1DM	190	102.3	1.46 (1.26, 1.68)	1.33 (1.15, 1.56)
T2DM	41	106.4	1.45 (1.06, 1.96)	1.35 (1.01, 1.79)
GDM	300	84.7	1.17 (1.04, 1.31)	1.09 (0.96, 1.23)
**Paternal diabetes**				
Unexposed	21,366	69.6	1.00 (Ref.)	1.00 (Ref.)
T1DM	122	78.7	1.10 (0.92, 1.31)	1.06 (0.88, 1.28)
T2DM	43	74.5	1.02 (0.76, 1.38)	0.90 (0.66, 1.24)

Note: CI, confidence interval; GDM, Gestational diabetes mellitus; HR, hazard ratio; T1DM, type 1 diabetes mellitus; T2DM, type 2 diabetes mellitus.
*Crude incidence rates per 100,000 person-years.
The HRs were pooled from 12 multiply imputed datasets. **Model 1** shows the unadjusted HRs. **Model 2** was adjusted for infant sex and birth year; maternal age at childbirth, parity, country of birth, educational level, cohabitation status, smoking, body mass index, alcohol use disorders, chronic hypertension, history of epilepsy, and psychiatric disorders; and paternal age at childbirth, history of epilepsy, and psychiatric disorders. Maternal and paternal diabetes were mutually adjusted for each other.
