# Supplementary material for: Intrauterine exposure to maternal diabetes and the risk of developing epilepsy in children: a national cohort study of 2.3 million children
Source: BMC Med. 2026 Feb 14;24:123. doi: 10.1186/s12916-026-04696-0 (PMC12930888; doi:10.1186/s12916-026-04696-0)
Supplement: Supplementary file 3 — Additional file 3. Supplementary Fig. S1-S4. Fig. S1–Study timeline from exposure assessment to outcome follow up. Fig. S2–Illustration of the negative control exposure design. Fig. S3–Log minus log survival curves and p-values from Schoenfeld residuals test by maternal diabetes categories. Fig. S4–Age-specific incidence rate of epilepsy in children with 95% confidence intervals [file 12916_2026_4696_MOESM3_ESM.pdf]

Additional File 3: Supplementary Figures

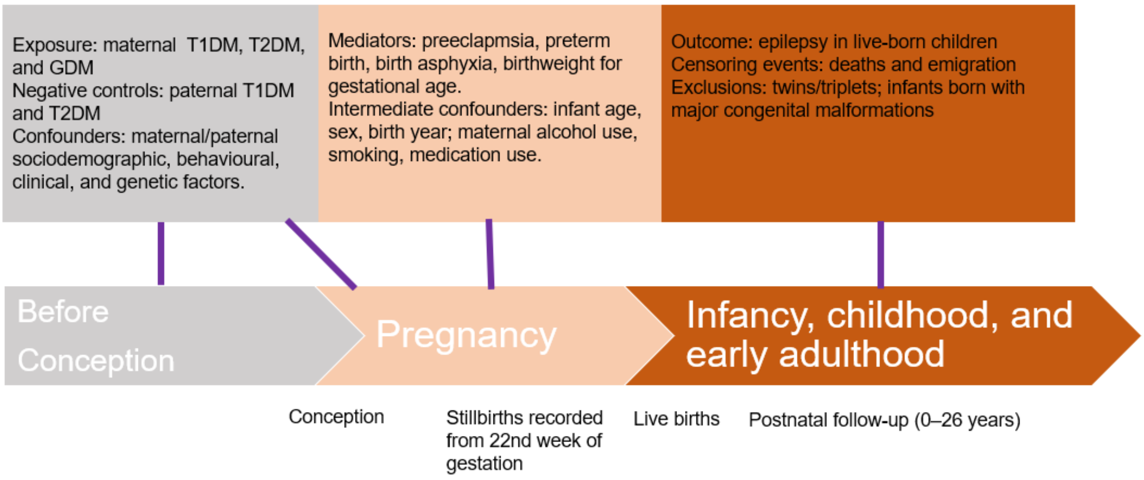

Note: GDM, Gestational diabetes mellitus; TIDM, Type 1 diabetes mellitus; T2DM, Type 2 diabetes mellitus.

Fig. S1. Study timeline from exposure assessment to outcome follow up

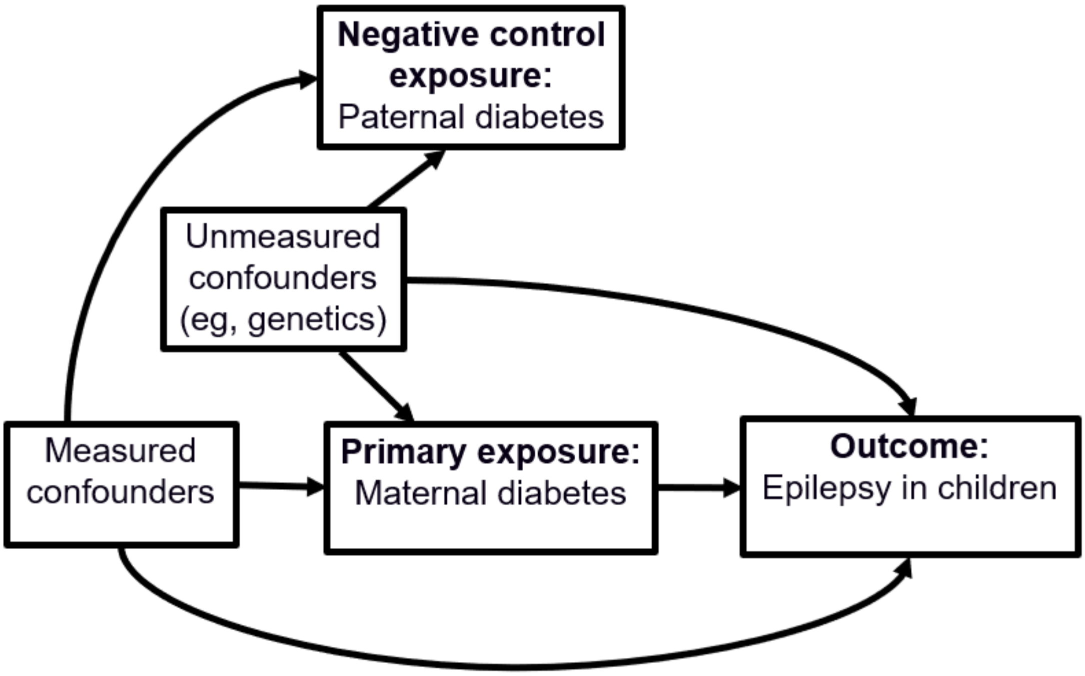

Note: Measured confounders include maternal socio-demographics (age, parity, country of birth, cohabitation status, educational level), maternal health (BMI, smoking, medication use, alcohol use disorder, chronic hypertension, psychiatric disorders, and epilepsy history), paternal health (diabetes, psychiatric disorders, epilepsy history, age), and child socio-demographics (age, sex, birth year). Unmeasured confounders may include genetics not captured by family history of epilepsy.

Fig. S2. Illustration of the negative control exposure design

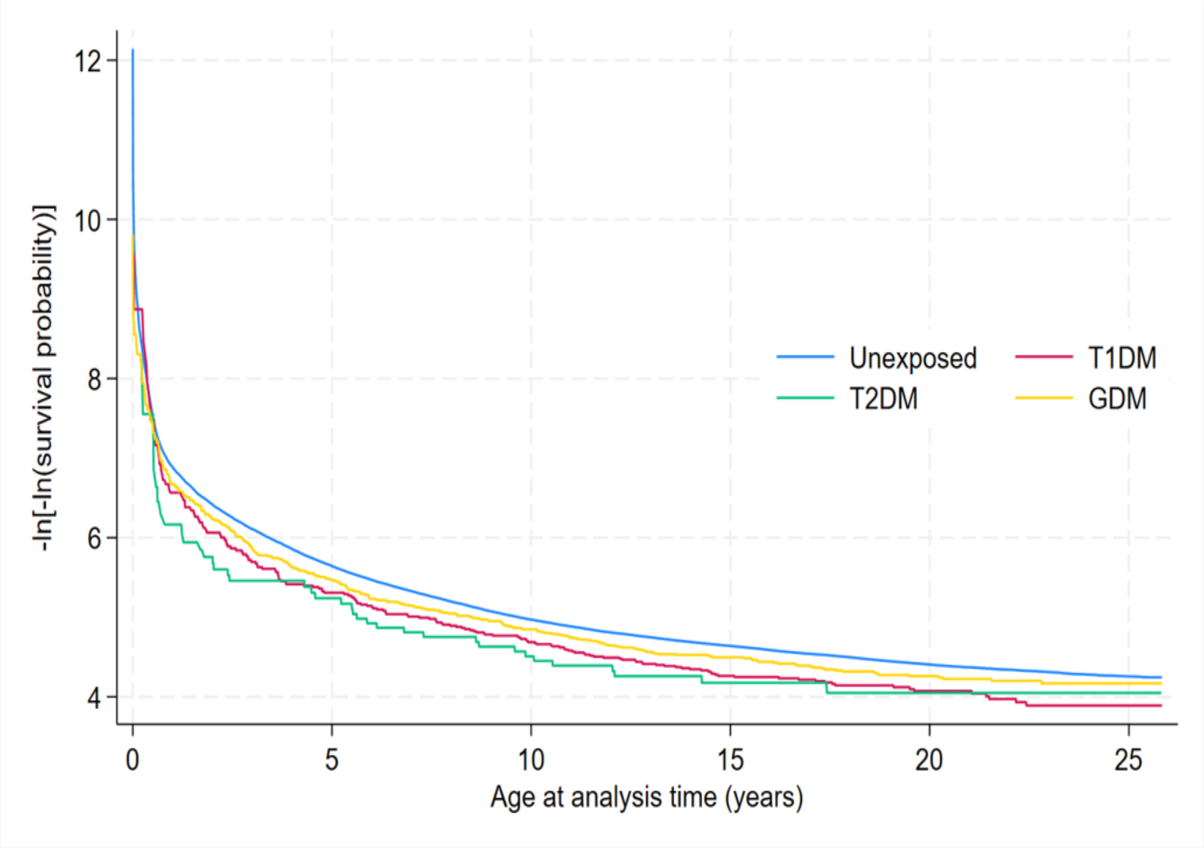

Schoenfeld residuals test for proportional hazards assumption:

P-value for T1DM: 0.697; P-value for T2DM: 0.470; P-value for GDM: 0.532; Overall p-value: 0.785.

Note: GDM, Gestational diabetes mellitus; TIDM, Type 1 diabetes mellitus; T2DM, Type 2 diabetes mellitus.

Fig. S3. Log minus log survival curves and p-values from Schoenfeld residuals test by maternal diabetes categories

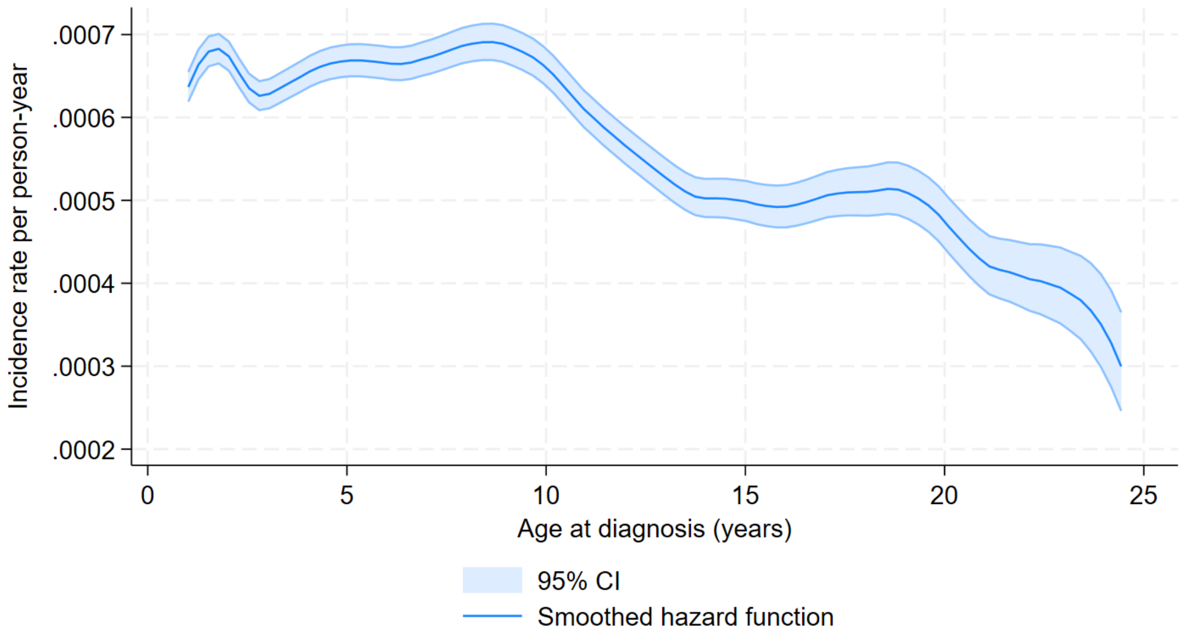

Note: The hazard function was smoothed using the Epanechnikov kernel function

Fig. S4. Age-specific incidence rate of epilepsy in children with 95% confidence intervals
